# Breast MRI during pregnancy and lactation: clinical challenges and technical advances

**DOI:** 10.1186/s13244-022-01214-7

**Published:** 2022-04-09

**Authors:** Noam Nissan, Ethan Bauer, Efi Efraim Moss Massasa, Miri Sklair-Levy

**Affiliations:** 1 Radiology Department, Sheba Medical Center, 5265601 Tel Hashomer, Israel; 2 Sackler Medicine School, Tel Aviv University, Tel Aviv, Israel; 3 Sackler Medicine School, New-York Program, Tel Aviv University, Tel Aviv, Israel; 4 Joint Medicine School Program of Sheba Medical Center, St. George’s, University of London and the University of Nicosia, Sheba Medical Center, Tel Hashomer, Israel

**Keywords:** Pregnancy, Lactation, Breast MRI, Pregnancy-associated breast cancer, PABC

## Abstract

The breast experiences substantial changes in morphology and function during pregnancy and lactation which affects its imaging properties and may reduce the visibility of a concurrent pathological process. The high incidence of benign gestational-related entities may further add complexity to the clinical and radiological evaluation of the breast during the period. Consequently, pregnancy-associated breast cancer (PABC) is often a delayed diagnosis and carries a poor prognosis. This state-of-the-art pictorial review illustrates how despite currently being underutilized, technical advances and new clinical evidence support the use of unenhanced breast MRI during pregnancy and both unenhanced and dynamic-contrast enhanced (DCE) during lactation, to serve as effective supplementary modalities in the diagnostic work-up of PABC.

## Key points

- Diffusion MRI may serve as a standalone modality during pregnancy.
- DCE MRI of the breast remains of significant value during lactation.
- Unenhanced DTI may increase PABC lesion conspicuity as compared with DCE.
- Non-fat suppressed T2 images can improve the delineating of non-mass DCIS lesions.
- Increased utilization of MRI may facilitate an earlier PABC diagnosis.

## Background

Pregnancy-associated breast cancer (PABC) is traditionally defined as breast cancer diagnosed during pregnancy, in the first year postpartum, or anytime during lactation [1], and typically represents a high-grade luminal b-like invasive ductal carcinoma [2]. Although it is a rare circumstance, occurring in 0.3 in 1000 pregnancies [3], breast cancer stands among the most common types of malignancies occurring during pregnancy and its incidence is on the rise in developed countries as more women delay childbearing [4, 5]. The diagnosis of PABC could be challenging because of the unique physiological changes that the breast undergoes [6], which may mask a concurrent malignant transformation both clinically and radiologically, while also dictating restrictions on the imaging work-up [7]. Ultimately, PABC is more likely to be diagnosed with an advanced disease than non-pregnant patients [8], and consequently, is associated with a poorer prognosis [9], being the most common cause of cancer-related mortality in gestational women and associated with a mortality rate that is 50% higher when compared to non-PABC [10].

Magnetic resonance imaging (MRI) and in particular, it’s workhorse sequence, dynamic contrast-enhanced (DCE) MRI, continues to serve in the mainstay of breast cancer diagnostic workup [11–13], and to expand in potential indications [14–16], owing to its high sensitivity for breast cancer detection and its unparalleled negative predictive value compared with conventional imaging [17]. In the general population, only the high costs and low availability are perhaps the main reasons to hold breast MRI from becoming widely used in screening. [18].

Nevertheless, despite its evident diagnostic superiority, inherent limitations of breast MRI surface during pregnancy and lactation, and as a result, restrictions are imposed on its utility [4, 19–21]. During pregnancy, DCE-MRI does not play a role in the diagnostic workup of the breast due to fetal safety concerns associated with gadolinium-based contrast agents [22], which are known to cross the placenta [23]. During lactation, gadolinium-based contrast is considered safe for administration [24]. Yet, breast DCE-MRI is considered controversial during lactation due to probable limited sensitivity caused by the increased characteristic background parenchymal enhancement (BPE), which may hinder suspicious finding [25–27].

In recent years, the advent of promising preliminary investigations and emergence of advanced MRI protocols, such as the increased clinical employment of unenhanced diffusion-based MRI techniques [28], has driven groups of radiologists and researchers to attempt to expand the role of breast MRI during pregnancy and lactation, with the hope to facilitate an earlier diagnosis of PABC. The aim of this pictorial article is to discuss and illustrate the latest developments of breast MRI during pregnancy and lactation. Variations in breast MRI manifestation due to the periodic physiological modifications are reviewed, as well as the MR imaging spectrum of common benign entities and PABC.

## Physiological changes of the breast

Throughout pregnancy, the breast undergoes a series of unique structural and functional alterations in preparation for its eventual biological role in lactation. Within this process, called lactogenesis, the mammary gland grows with developed glandular tissue at the expense of shrunken adipose and connective tissues [29]. Regulated by key hormones, lactogenesis is composed from two stages, which is necessary for the breast in order to synthesize and secrete milk [30]. Secretory initiation takes place in the second trimester of pregnancy. In the postpartum period, secretory activation, the second stage of lactogenesis, begins and is followed with milk secretion which is triggered by the fall of progesterone blood levels [31]. The colostrum is temporarily enriched with protein and electrolytes and following several days of breastfeeding, turns into a mature, lipid-rich, and stable mother’s milk [32].

**Fig. 1:**
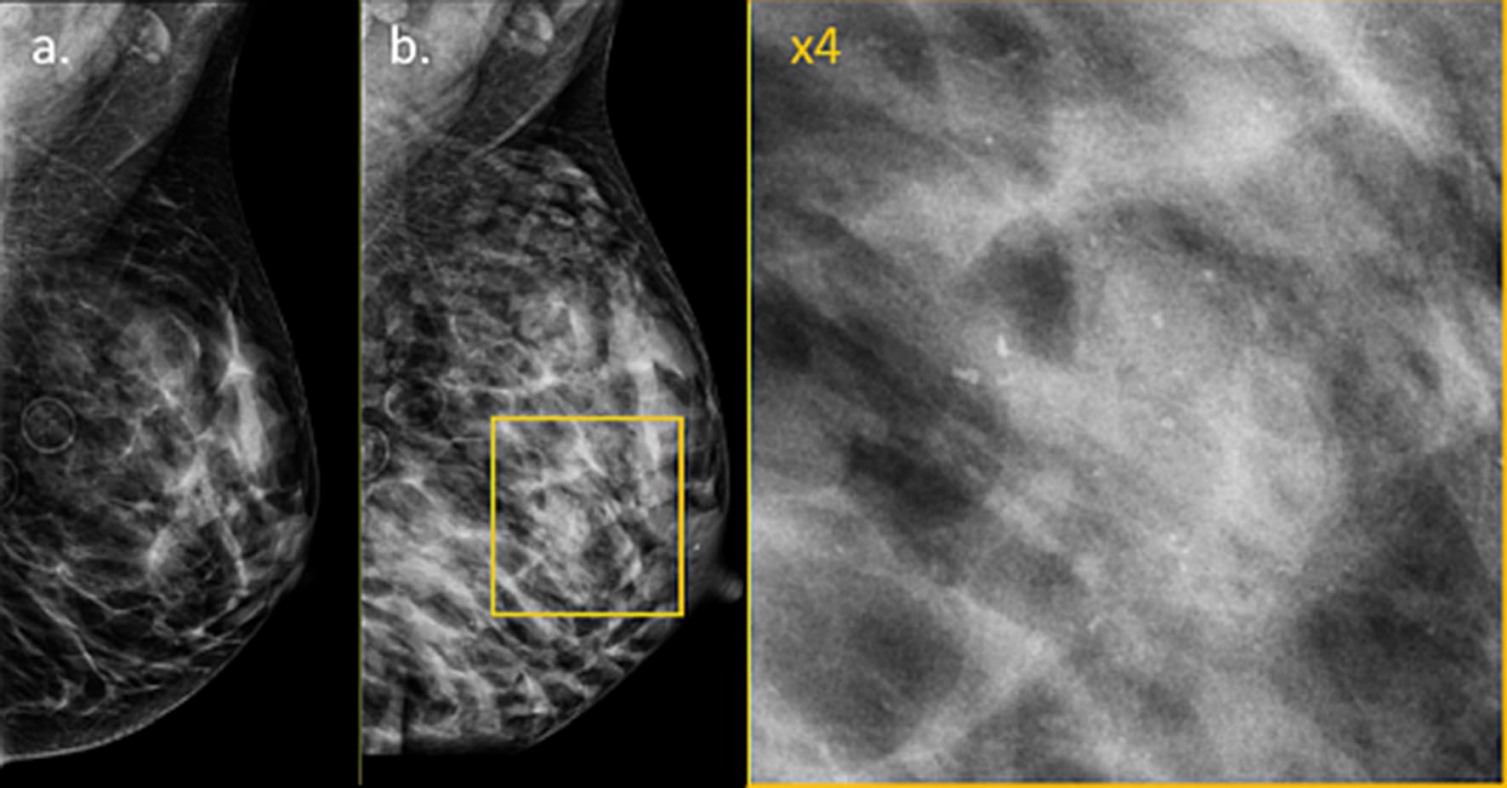
Changes in mammographic density and lactation-associated microcalcifications. Left breast medio-lateral-oblique view mammograms of the same patients, performed 2 years apart, prior to pregnancy (**a**) and during lactation (**b**) are presented, demonstrating the marked increase in breast volume and mammographic density associated with pregnancy and lactation. Additionally, new onset of lactation-associated microcalcifications was depicted globally on both breasts (**b** and zoomed image)

Both clinical and radiological evaluation of the breast are influenced by the physiological changes during pregnancy and lactation. Clinically, breast examination can be challenging due to the enlarged size of the breasts, their tenderness, and especially their harder, more nodular consistency [33]. Imaging-wise, each of the various radiological modalities is hampered by the various changes in the breast properties. Owing to its harmless nature and excellent utility in focal evaluation of palpable findings [34], there is consensus that US represents the most appropriate and thus, the first-line imaging modality for breast evaluation during pregnancy and lactation [35]. The role of mammography is relatively diminished during pregnancy and lactation due to the increased mammographic density of the breast parenchyma and concerns related to radiation exposure for the fetus [19]. It should be mentioned though, that in many centers, mammography is considered generally safe during pregnancy and lactation, since the radiation dose from a bilateral two view mammogram is < 3 mGy per view, equivalent to 7 weeks of background radiation [36]. The increased mammographic density (Fig. 1) may well reduce the sensitivity of screen-detected tumors [37], although mammography may still be useful in the detection of suspicious micro-calcifications. Therefore, mammography serves as an adjunct to US [27]. During lactation, patients are advised to nurse or pump immediately before undergoing mammography in order to decrease parenchymal density related to retained milk [6]. Rarely, mammography can also exhibit a unique form of scattered micro-calcifications, secondary to gestational (pregnancy) or secretory (lactation) hyperplasia, which may add further complexity for mammographic evaluation (Fig. 1) [38, 39].

**Fig. 2:**
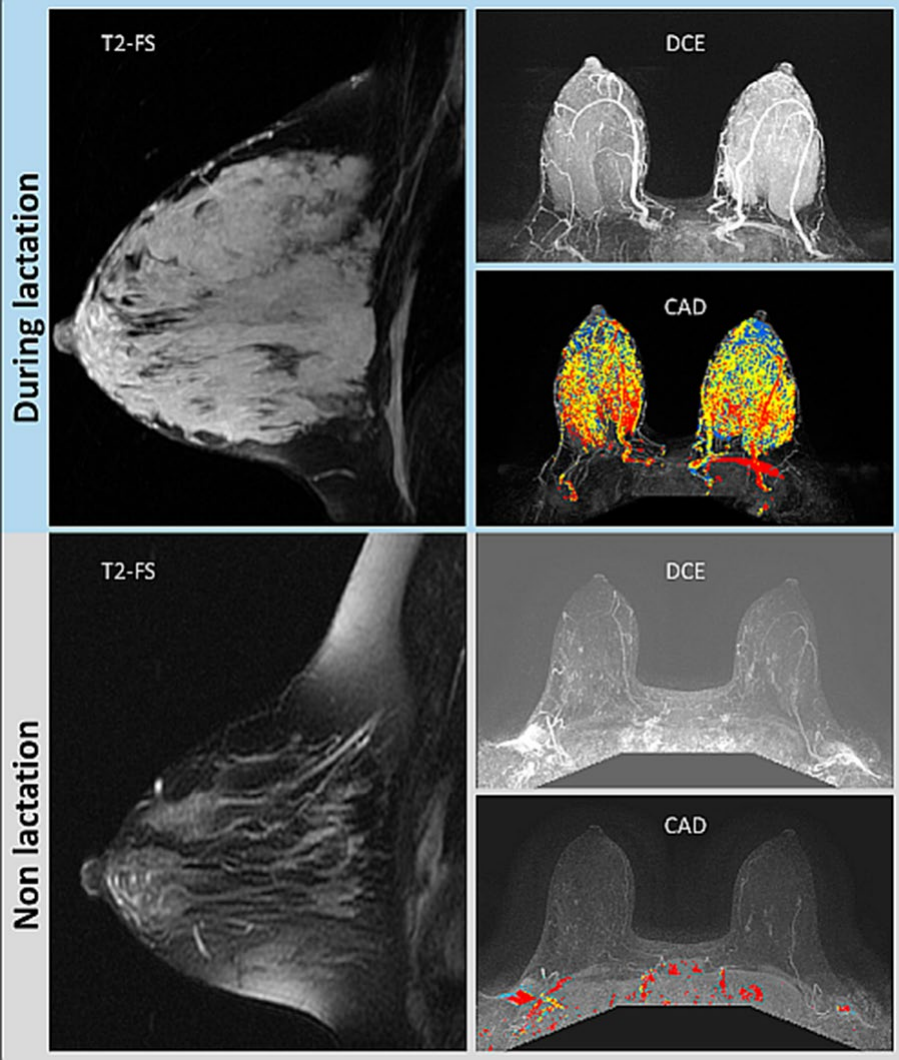
Changes of breast MRI properties during lactation. MR images of a BRCA carrier scanned twice at the age of 37, while lactating for 4 months (upper panel), and 2 years afterwards, post-weaning (lower panel) are presented. The sequential examinations demonstrate the marked changes in the breast composition and vascular properties between the two periods. A rich fibroglandular tissue, which is exhibited during lactation, reduces dramatically post weaning. Respectively, the breast perfusion reduces considerably, from BPE 3 to BPE 1, as shown on DCE subtraction MIP images, as well as on DCE CAD maps. Add DCE MIP

These physiological changes of the breast are also reflected on the various MRI pulse sequences. Since contrast enhanced breast MRI scans are not performed during pregnancy, reports on breast MRI of pregnant patients have been limited to examinations performed prior to elected abortion [40] or using unenhanced protocol [41]. Breast MRI studies of the lactating breast are more common and include both DCE, as well as unenhanced sequences [40, 42–52]. The main MRI features of the breast during pregnancy and lactation are increased fibroglandular tissue [47] and increased vascularity, which is manifested by marked BPE [40, 42, 43, 49–52] and results in false positive coloring on DCE computer aided diagnosis (CAD) mapping (Fig. 2).

Further characterization of the lactating breast has been afforded by additional MRI sequences. On MR spectroscopy, a total choline peak, an established breast cancer biomarker [53], was evident in exams of most healthy lactating volunteers, thus limiting its clinical role in this population [44]. On diffusion-weighted imaging (DWI), the measured apparent diffusion coefficient (ADC) of the lactating breast was found to be decreased relative to normal values among premenopausal, non-lactating, and healthy volunteers. This phenomenon is most likely due to the increased viscosity of the lipid-rich milk [45], although the ADC is still higher than the malignant spectrum of values [44, 45, 47, 48].

**Fig. 3:**
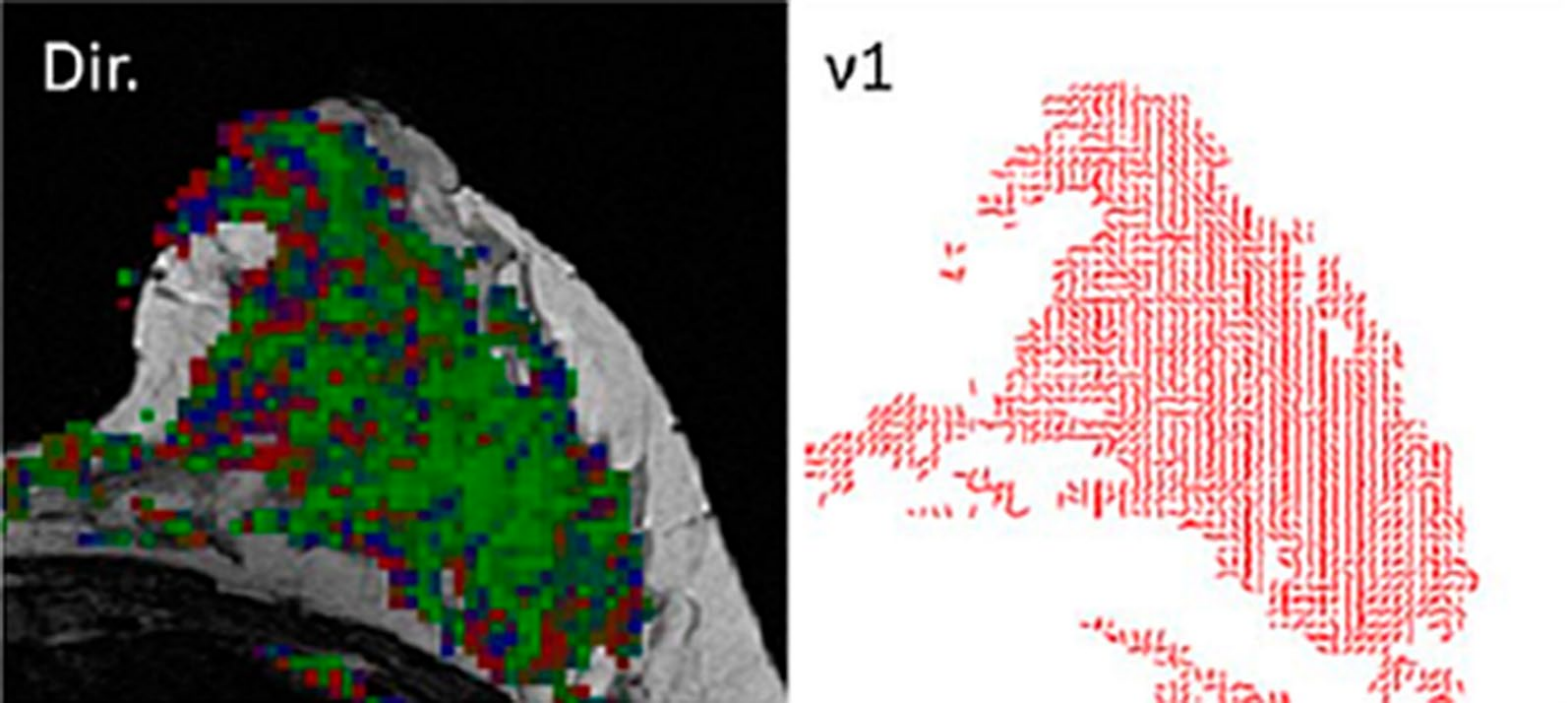
Characterization of the lactating breast using DTI. DTI-derived direction and vector maps of a 30-year-old healthy lactating volunteer are presented. Axial images overlaid on anatomical non-fat surpassed T2-weighted images at the height of the nipple are present. The direction map represents the direction of the 1st principal eigenvector in a three color code: red: right <<>> left; green: head <<>> feet; blue: anterior <<>> posterior. The vector map presents in red sticks the direction of the 1st principal eigenvector ν1. Note: The lactating breast exhibits dominant directional diffusion along the anterior–posterior axis, reflecting the structure of milk ducts heading from the base of the breast towards the nipple, while the vector map portray linear successive “duct-like” structures towards the nipple. Adopted from Nissan et al. Proc. Intl. Soc. Mag. Reson. Med. 22 (2014)

Advanced diffusion MRI models were also used to investigate the unique features of the breast during pregnancy and lactation. Intra-voxel incoherent motion (IVIM), a bi-exponential diffusion model, is based on acquiring multiple diffusion weightings in the fast and slow regimes in order to separate the fast perfusion-based “pseudo-diffusion” component from the slow diffusion process [54]. Using IVIM analysis, the lactating breast, as anticipated, has shown to exhibit increased perfusion fraction [48], due to the pronounced vascularity of the breast parenchyma [42] and the high metabolic demand during breastfeeding [55]. Another approach could be found in diffusion tensor imaging (DTI), which is based on applying diffusion gradients to characterize tissue microstructure. These gradients go in multiple directions in order to map spatial information of the diffusion hindrance and restriction that goes beyond cellular density [56]. DTI properties among healthy, pregnant examinees resembled the measurements among non-pregnant, premenopausal examinees, with relatively high values of diffusivity, as expected for dense breasts [41]. Besides decreased diffusivity, DTI studies of the lactating breast also reported reduced anisotropy [45, 46], probably owing to the physiological transient increase in the diameter of the lactiferous ducts [57]. Furthermore, DTI has enabled the characterization of the underlying ductal-tree architecture of the lactating breast, as demonstrated by the diffusion Eigen-vectors mapping. This is clearly illustrated by the predominance of diffusivity directed to the nipple with “duct-like,” linear, and branching vectors of the first eigenvalue [30, 46, 58] (Fig. 3).

## Benign breast disease of pregnancy and lactation

Benign entities account for the vast majority of findings among patients presenting with a palpable breast mass during pregnancy and lactation [6]. In a study that evaluated the diagnostic workup of 164 lesions among pregnant, lactating, and postpartum women, Robbins and co-authors reported that most of the cases appeared during lactation (65%), and breast cancer accounted for only 2.4% of cases (4/164), even though cancer constituted 10% of the eventual biopsies [59]. Benign conditions, however, are more common and are either the same as those observed in non-pregnant women [60] or breast abnormalities distinctive for pregnancy and lactation [61]. Examples of these mimickers include, though not exclusively, galactocele, lactating adenoma, fibroadenoma, duct ectasia, mastitis, and abscess [62], along with common contemporary mimickers that affect breast imaging, such as vaccination induced lymphadenopathy [63]. Their presentation, with focus on their MRI characteristics, will be discussed below. In addition, a summary of the typical MRI features of the common breast lesions during pregnancy and lactation is provided in Table 1.

**Table 1:** A summary of the MRI features of common breast entities during pregnancy and lactation

Entity	MRI features
Galactocele	Non-fat-suppressed T1-weighted and fat-suppressed T2-weighted images could determine the diagnosis among lactating patients with a cystic lesion including a thin septa, heterogeneous content, and fat-fluid level
Lactating adenoma	Morphology-wise resembles a fibroadenoma-like lesion; a well circumscribed mass, containing hypointense septa, causing mass effect on adjacent parenchyma and the main galactiferous ducts of the NAC
	On DCE, a benign kinetic curve of persistent enhancement may appear
Fibroadenoma	Morphology-wise, usually exhibits a benign shape on unenhanced sequences, including a sharp contour without signs of infiltration
	On DCE, typically exhibits benign patterns of a persistent kinetic curve
Duct-ectasia	Fat-suppressed T1 and T2-weighted images may display an enlarged ductal structure, depending on if its content is composed of protein or fluids, respectively
	A unilateral duct dilatation may be an indicator of malignancy and could enhance on DCE, therefore requiring a further diagnostic work-up
Mastitis and Abscess	MRI is not indicated during acute mastitis; however, if symptoms persist despite optimal treatment, MRI may be performed to rule out an inflammatory breast cancer
	On DCE these two differentials may exhibit an overlapping suspicious features, thus clinical correlation and tissue sampling may be indicated
PABC	*During pregnancy*—unenhanced DWI/DTI could determine the diagnosis
	*During lactation*—DCE is of value, despite BPE limitations. DWI/DTI can improve tumor conspicuity. Hypo-intensity on T2 weighted images can assist in delineation of NME lesion, like DCIS
	*Post-weaning*—BPE drops, and DCE utility returns to optimum

### Galactocele

**Fig. 4:**
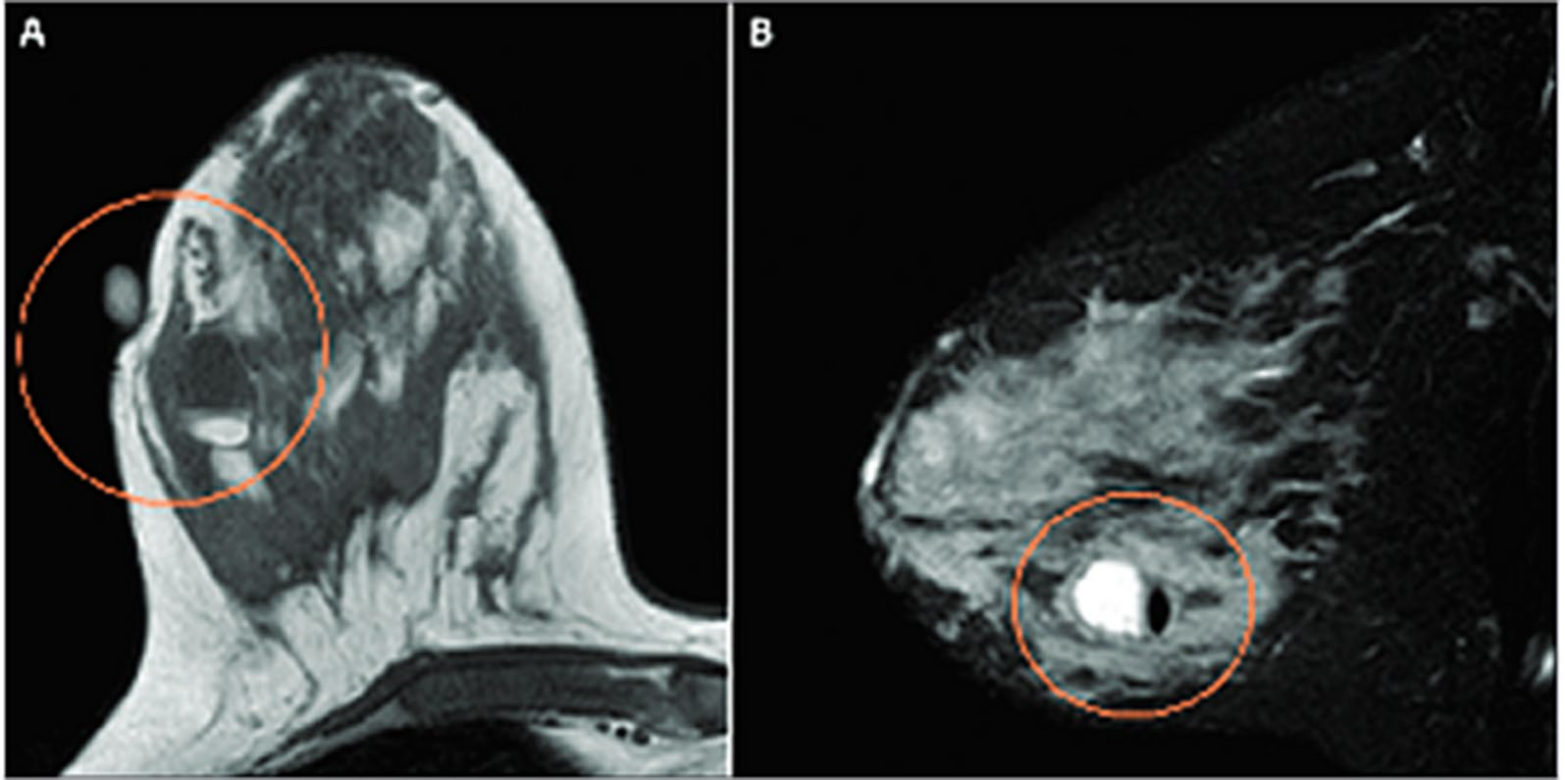
Galactocele. Non Fat-suppressed axial T1-weighted (panel **A**) and sagittal fat-suppressed T2-weighted (panel **B**) images of lactating patient with galactocele are presented. *Note*: a cyst with thin septa, heterogeneous content, and fat-fluid level is exhibited and compatible with galactocele diagnosis. Reproduced with permission from Radiologia Brasileira  [19]

A galactocele, a Greek term meaning “milky pouch,” is a milk collection retained within the fibroglandular tissue because of duct obstruction. This etiology usually regresses spontaneously on follow-up and is the most common benign breast mass among lactating patients [64]. Characterized with a cyst-like formation, a galactocele is often surrounded by a fibrous capsule with variable luminal morphology depending on the distribution of its contents: fat, protein and fluid [61]. Mostly encountered after cessation of breast-feeding, galactoceles can also be present earlier, occasionally even in the third trimester of pregnancy [6]. Similar to other pregnancy-associated breast lumps, the typical clinical manifestation is a painless, palpable mass, arising upon breastfeeding cessation [60]. From the imaging perspective, galactocele is mostly described according to its sonographic appearance [65]; usually as round or oval in shape, with variable echogenicity which most likely increases as the lesion ages and a characteristic fat-fluid level [66]. MRI features of galactocele are hardly described in the literature, as US is sufficient for its diagnostic work-up. Recently, Rosas et al. provided MR images showing a cyst with a thin septa, heterogeneous content, and fat-fluid level, which is compatible with the diagnosis [19] (Fig. 4).

### Lactating adenoma

**Fig. 5:**
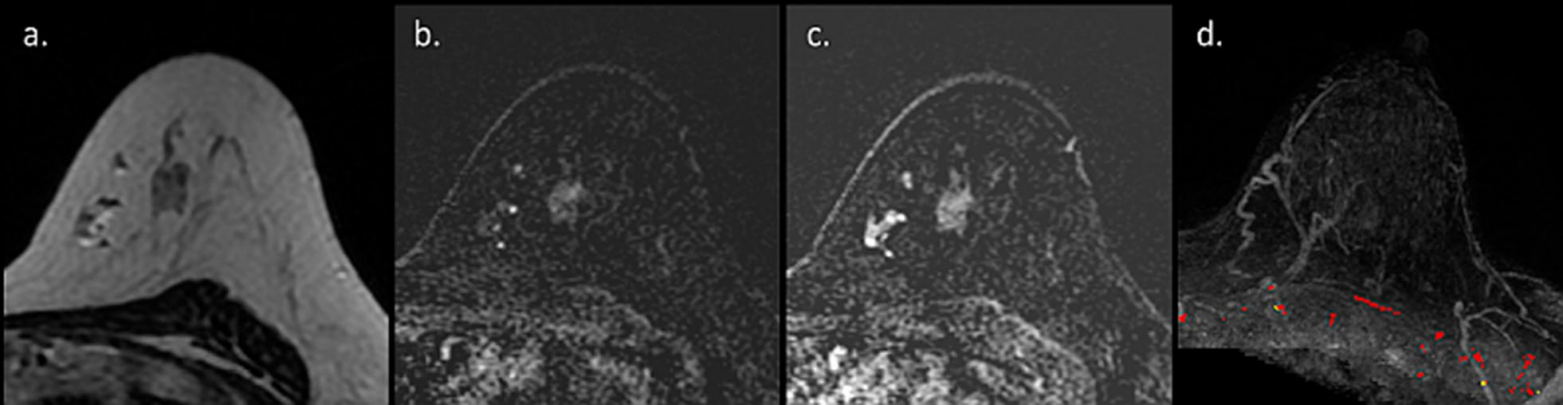
MRI of lactating adenoma. MR images of patient presented with palpable mass on the left breast 2 months after breastfeeding cessation are presented. T2 weighted image showed a hypo-intense mass (**a**), which exhibits a gradual enhancement and early and late DCE (**b**, **c**), below the signal intensity threshold for suspicious findings on CAD (**d**). Despite these benign features, the patient underwent vacuum-assisted biopsy which revealed lactating adenoma

Lactating adenoma represents a benign stromal alteration with a tendency to regress upon breastfeeding cessation [67]. Lactating adenoma is the most prevalent breast lesion occurring during pregnancy, usually appearing during the third trimester or during lactation, as a painless, palpable, and mobile breast lump [68]. Typical US features of lactating adenoma favor a benign mass, including a solid, ovoid, well-defined, and wider-than-taller lesion with homogeneous and hypoechoic appearance alongside posterior acoustic enhancement [69]. On MRI, lactating adenoma has been described as fibroadenoma-like; a well circumscribed mass, containing hypointense septa, causing mass effect by displacing the adjacent normal breast parenchyma and the main galactiferous ducts of the nipple-areolar complex [70]. Herein, we present another representative MRI case of a biopsy-confirmed lactating adenoma. Our findings suggest that lactating adenoma may exhibit benign features of enhancement kinetics on DCE MRI (Fig. 5).

### Fibroadenoma

**Fig. 6:**
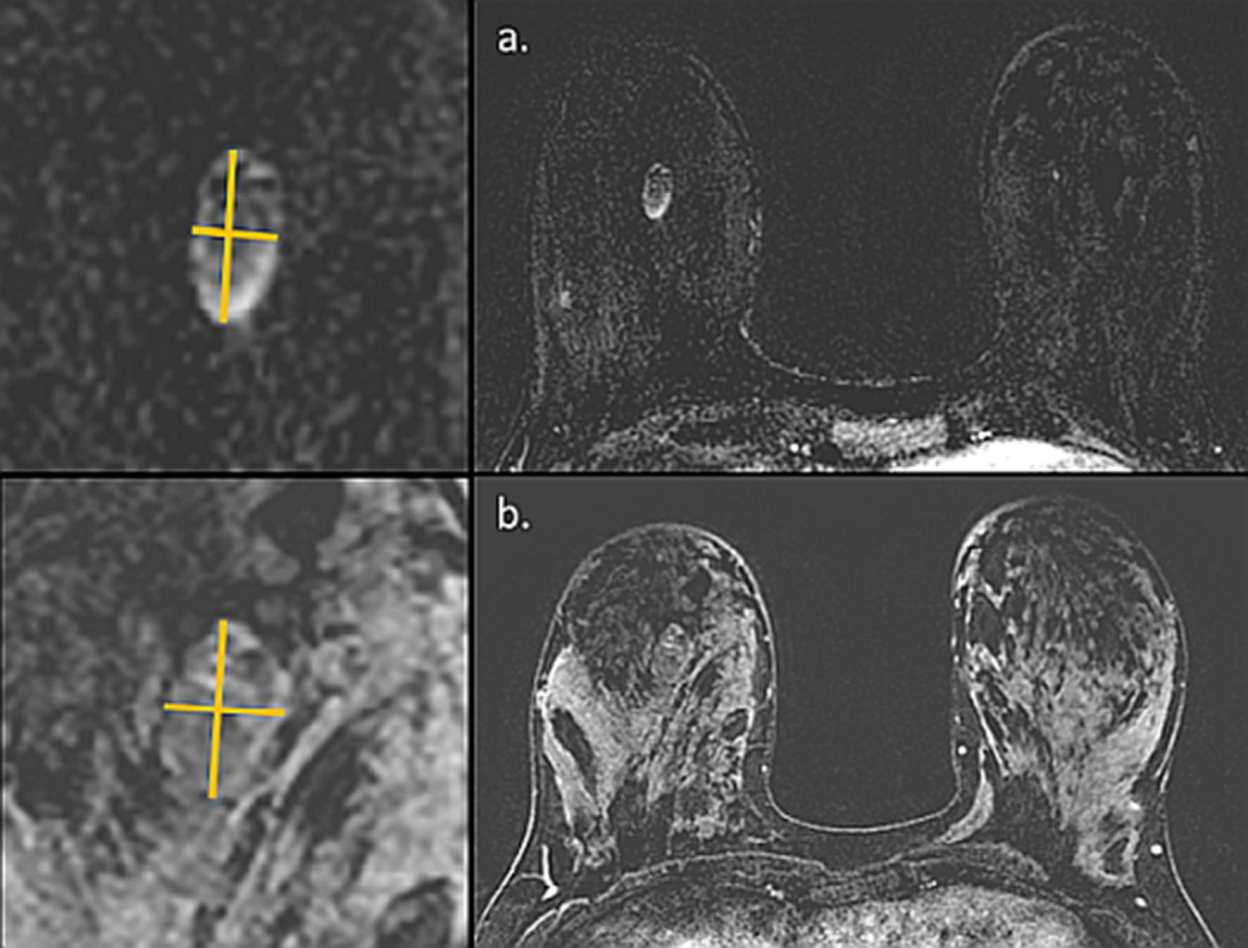
Growing fibroadenoma during lactation. DCE-MRI subtracted images of a 26 years BRCA 1 carrier scanned twice within 18 months of routine surveillance, before conception and during lactation are presented. On baseline MRI (**a**), a 1.3 cm well-defined enhancing oval mass is visible on top of minimal background parenchymal enhancement (BPE 0). Yet, on follow-up during lactation, a 1.8 cm is hardly visible on DCE, due to the marked physiological BPE (grade 3) (**b**). The lesion enlargement and a personal history of phyllodes tumor prompt a US-guided biopsy which reassured fibroadenoma histology

Fibroadenomas are composed of epithelium and stroma and account for the most common benign tumor detected in young females [71]. Interestingly, before pregnancy, fibroadenomas may remain latent and asymptomatic until becoming clinically apparent as a new-onset palpable mass after hormonally stimulated growth [6]. Clinically, fibroadenomas, which are often multiple and bilateral, usually present as a painless firm, mobile, and rubbery mass. Less frequently, fibroadenomas may experience a tremendous growth spurt, resulting in central infarction, and then becoming tender [72]. On mammography, fibroadenomas often appear as a well-defined round or oval mass which may also exhibit pathognomonic benign calcifications, making a further imaging work-up unnecessary [73]. On US, fibroadenomas among pregnant or lactating women is the same as among the general population, exhibiting a circumscribed, wider-than-taller oval or round mass [74]. Infarcted or complexed fibroadenomas may show suspicious features such as irregular margins and internal cystic changes that warrant biopsy [75]. On MRI, fibroadenomas usually exhibit a benign morphology on unenhanced sequences, including a sharp contour without signs of infiltration [76]. Additionally, they exhibit benign DCE patterns such as a persistent kinetic curve [77] and a high extracellular volume fraction with low to moderate microvascular permeability [78]. A representative case of a growing fibroadenoma is given in Fig. 6.

### Duct ectasia

**Fig. 7:**
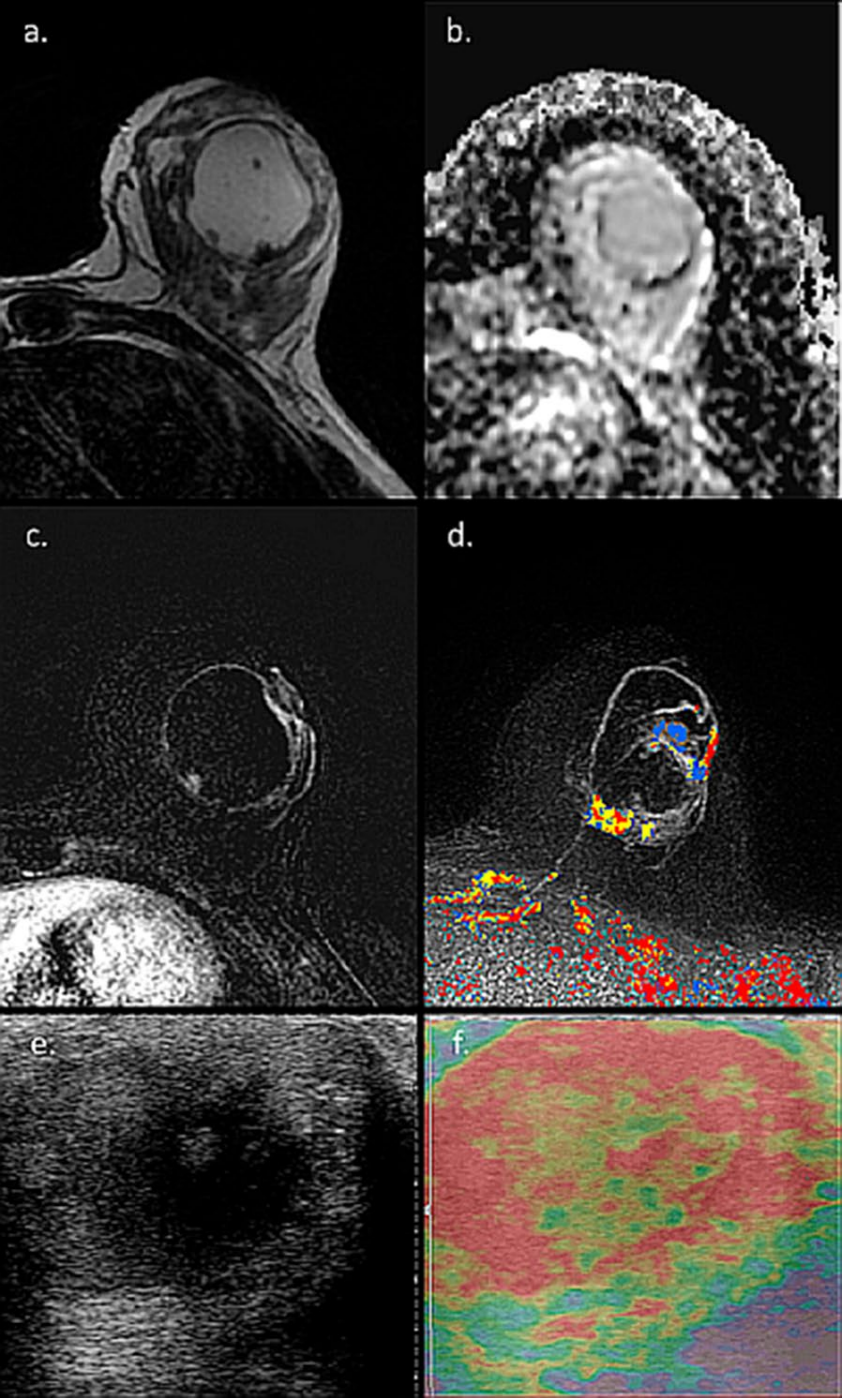
MRI of duct ectasia. MRI and US images of 40-year-old lactating patient with palpable mass on the left breast are presented. Initial work-up included US which revealed a 4 cm cystic mass with thick boundaries, with relative mixed cystic areas (**e**) and stiffness on static elastography (**f**). On MRI, T2-weighted image reveals a high signal cystic structure (**a**), without internal restriction on ADC map (**b**), though with enhancement of its walls on DCE and CAD (**c**, **d**). Finally, US-guided biopsy ruled out malignancy with duct ectasia diagnosis

Duct ectasia of the breast is among the benign processes that may affect the nipple-areolar complex during pregnancy and lactation [79]. The clinical course of duct ectasia ranges from asymptomatic to symptoms such as nipple discharge, nipple retraction, a palpable mass, and mastalgia [80]. Depending on the degree of dilatation as well as the mammographic density, duct ectasia may be visible at mammography as dense tubular structures converging on the nipple-areolar complex [81]. Sonographically, it appears as anechoic, smooth-walled, and branching structures that taper peripherally [82]. On MRI, the ductal structures may be visible on fat-suppressed T1 and T2-weighted images depending on if its contents are composed of protein or fluids, respectively. Despite being regarded as benign, a unilateral duct dilatation may be an indicator of malignancy and hence, the importance of its diagnostic work-up [83]. An illustrative case of duct ectasia mimicking malignancy is shown in Fig. 7.

### Mastitis and abscess

**Fig. 8:**
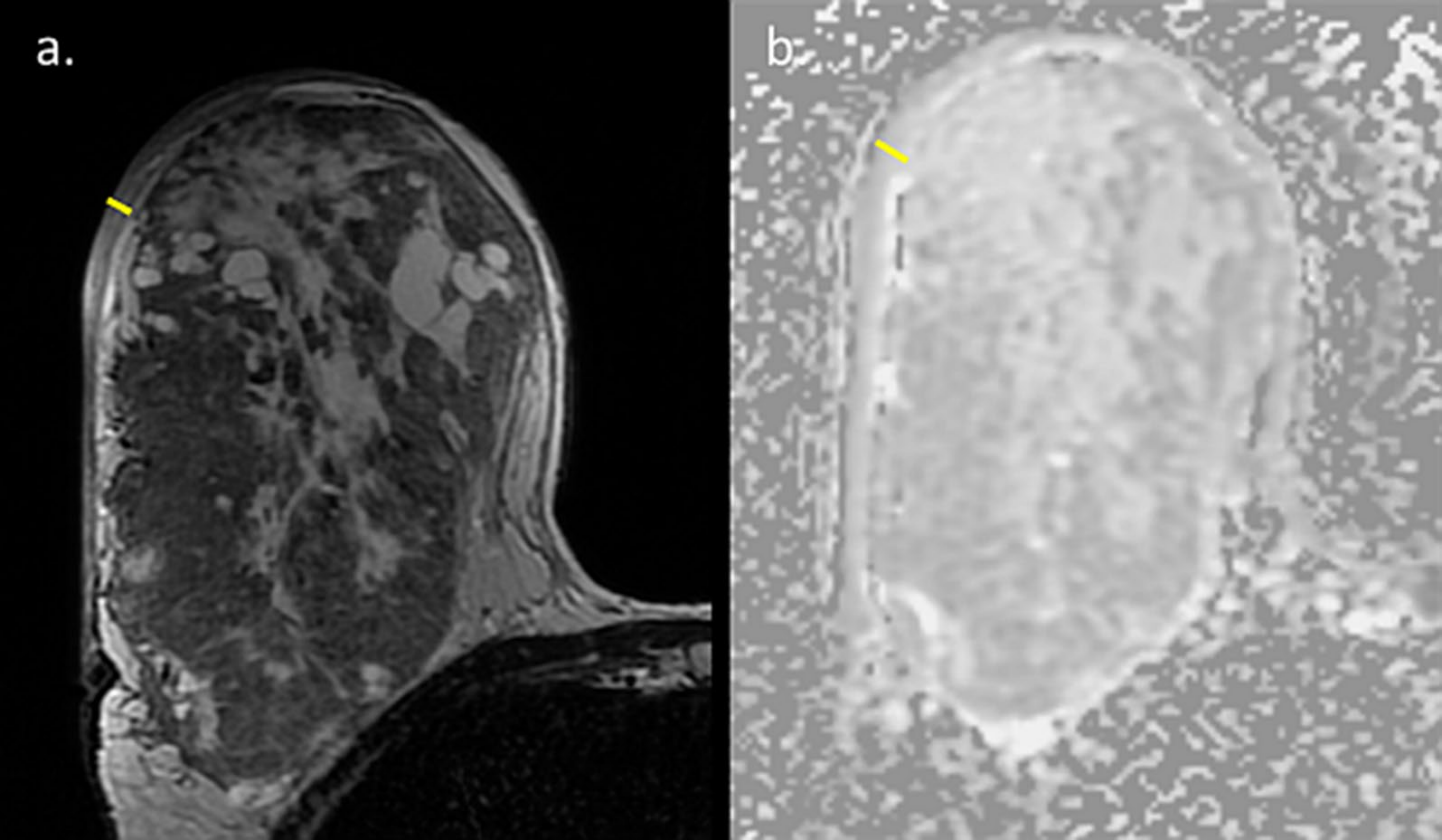
Mastitis. Unenhanced MR images of 37-year-old pregnant patient with refractory mastitis are presented. The patient presented with breast edema, erythema and pruritus and blinded subcutaneous punch biopsies revealed adenosis on pathology. Because of continuous symptoms despite treatment, we were requested to perform MRI without contrast injection to rule out underlying inflammatory carcinoma. T2 weighted image revealed thickened skin (**a**) (yellow line), while no focal restriction was noted on ADC map (**b**)

**Fig. 9:**
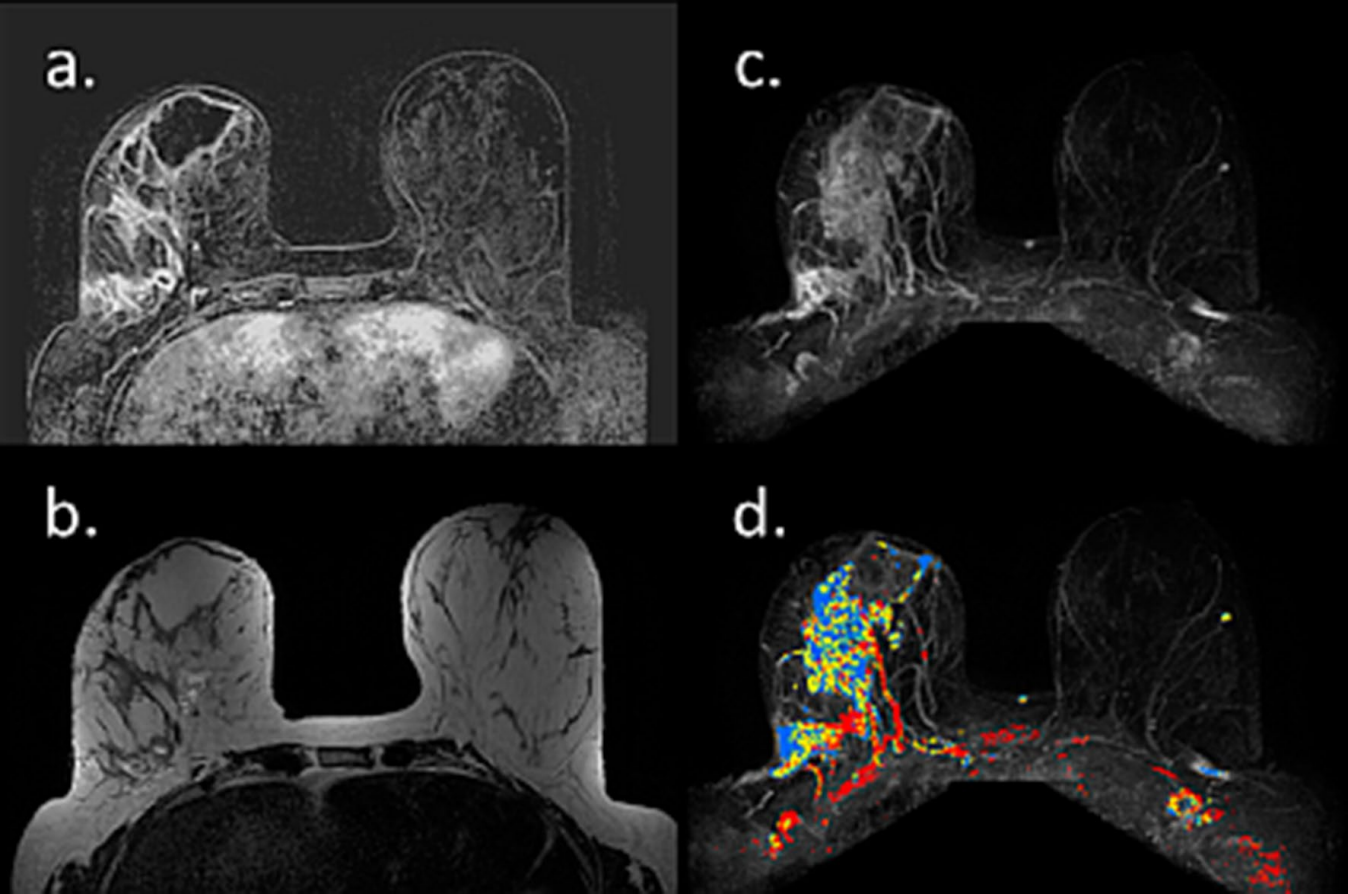
Abscess. MRI of 31-year-old patient with a history of recurrent breast mastitis and abscesses during several separate lactation periods is presented, this time in aspiration-confirmed breast abscess post weaning. Subtracted DCE (**a**) MIP (**c**) and CAD (**d**) reveal large rim enhancing regions with high T2 signal (**b**), compatible with an abscess

Mastitis is a common infectious condition that may affect up to one-third of lactating women [84] and is among the leading medical causes of premature breastfeeding cessation [85]. Among the most common complications are mastitis that are abscesses with a purulent collection. Its pathophysiology is thought to be related to transmission of oral bacterial from the infant to the mother’s lactiferous ducts. Maternal risk factors that were identified include previous mastitis during breastfeeding, cesarean section, breast trauma, latch problems, milk overproduction, blocked duct, and more [86]. Clinically, mastitis presents with focal mastalgia, edema, and erythema which may be accompanied fever and elevated blood test inflammatory markers. Focused US is indicated to rule out abscess when the infection is refractory to antibiotics, or for therapeutic guided-aspiration of the abscess [87]. Sonographically, it typically is characterized by an area of fluid collection with thin septations or debris, thickened walls, uncircumscribed margins and posterior acoustic enhancement [60]. With that regards, another related entity worth mentioning is granulomatous mastitis (GM), a rare benign inflammatory breast disease that affects mostly women of childbearing age with a history of breastfeeding and may mimic both abscess and carcinoma [88]. Breast MRI is not indicated during acute mastitis; however, when mastitis symptoms persist despite well-managed medical treatment, MRI may be performed. The main differential diagnosis of exclusion is inflammatory breast cancer [89], notwithstanding overlapping enhancement features of the two entities [90]. Herein, we present two cases in which MRI was utilized during for mastitis evaluation (Fig. 8) and abscess monitoring (Fig. 9).

## Pregnancy-associated breast cancer (PABC)

### Pregnancy

**Fig. 10:**
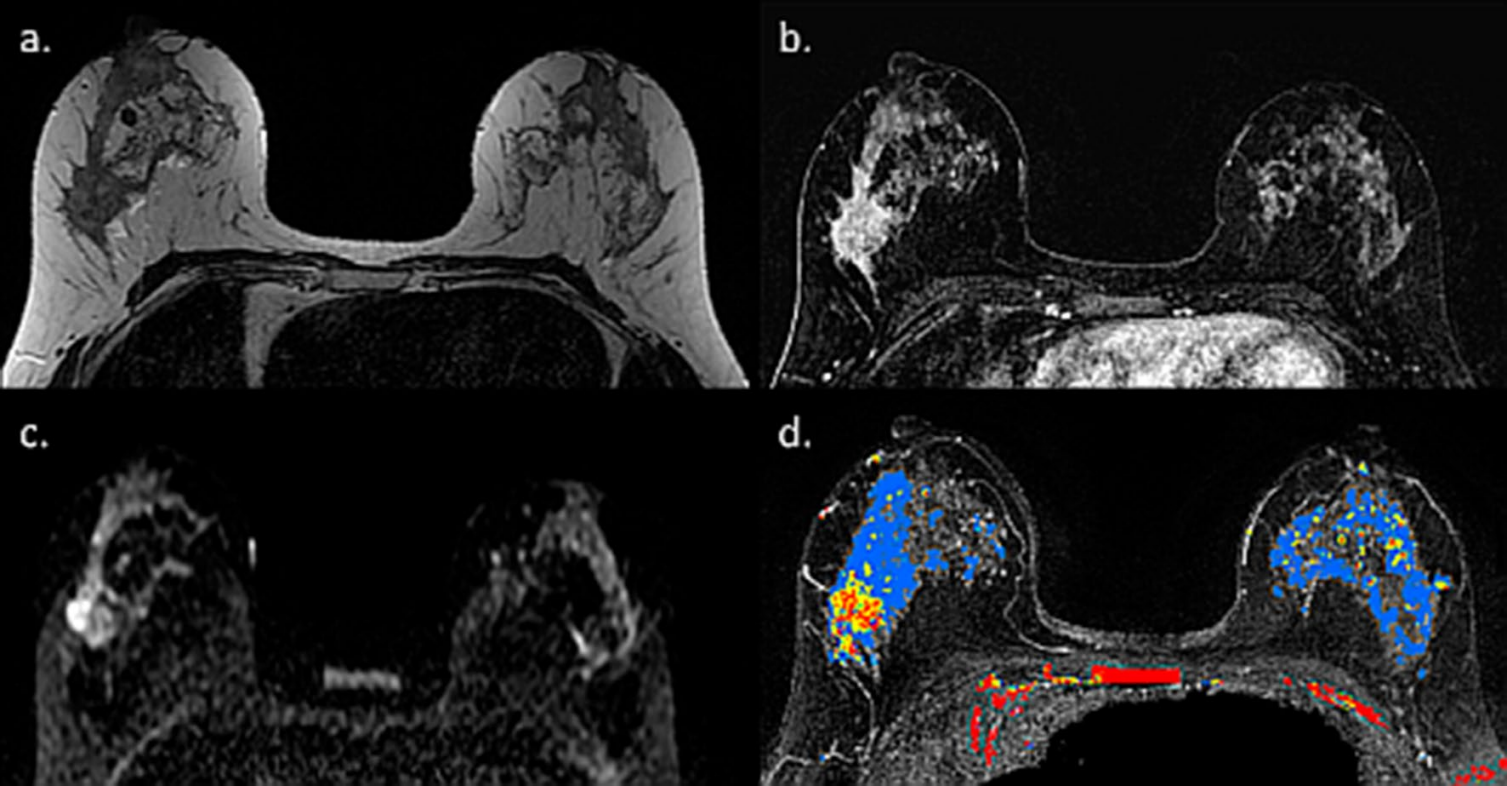
MRI of PABC during pregnancy prior to elected abortion. Pre-operative MR Images of a pregnant patient with newly diagnosed IDC are shown. T2 (**a**), subtraction DCE (**b**), DWI (**c**) and CAD (**d**) reveal extensive lesion on the right breast, on top of marked BPE on both breasts, as well false positive bilateral CAD coloring secondary to the increased vascularity

During pregnancy, breast DCE-MRI is contraindicated due to the increased risk of a broad set of rheumatological, inflammatory, or dermal conditions, as well as stillbirth or neonatal death, associated with gadolinium-based contrast agents used during the MRI [22]. The lone report on breast DCE-MRI during pregnancy was composed of PABC patients who elected to undergo abortion [40]. Despite the lack of supportive evidence for improved maternal outcomes for pregnant breast cancer patients undergoing therapeutic abortion [91], an elective abortion remains frequent in patients diagnosed in the first trimester [92]. In these patients, DCE-MRI can aid in improved pre-operative assessment, providing additional diagnostic information regarding tumor size, extent of disease and contralateral involvement compared to mammography and US, in up to 28% of cases [40]. A representative MRI of a pregnant breast cancer patient who elected to undergo abortion is given in Fig. 10, showing the tumor extent superimposed on the notable pregnancy-associated BPE.

**Fig. 11:**
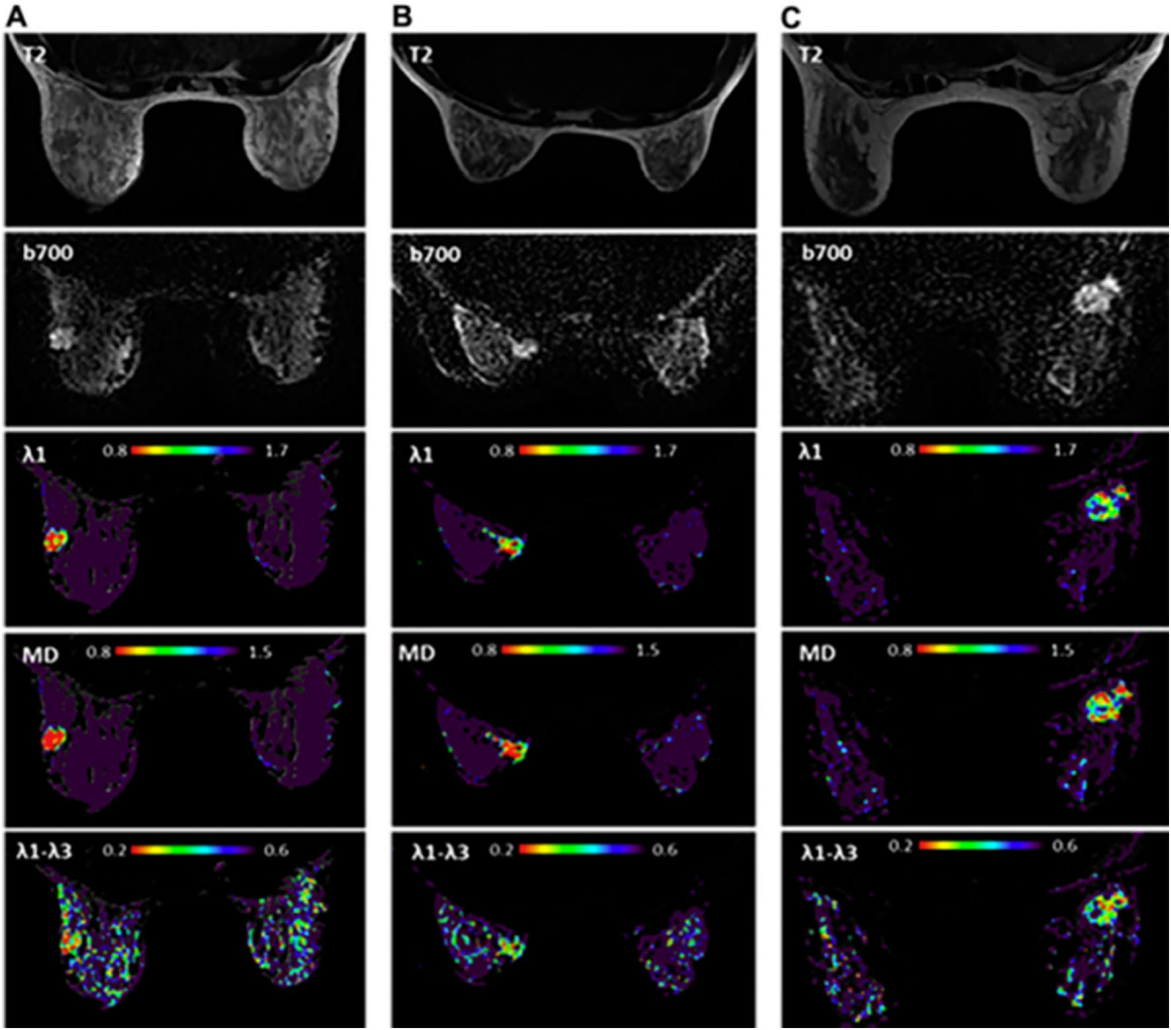
Unenhanced diffusion MRI during pregnancy. T2, DWI, and DTI parametric maps of three PABC patients (**A**–**C**). T2-weighted, DWI and DTI-derived diagnostic parametric maps of *λ*1, MD, and *λ*1–*λ*3 of three patients with newly diagnosed IDC are presented. The lesion appears bright on DWI (b 700 s/mm^2^). Using the parametric threshold, the lesion could be easily depicted on l1, and MD maps, as well as on l1–l3 map, compared with the measurements in the normal tissue. Reproduced with permission from Journal of the American College of Radiology

Moreover, the first attempt to utilize unenhanced diffusion MRI as a standalone modality for pregnant patients at high risk or with newly diagnosed PABC was recently reported [37]. This work demonstrated the feasibility and tolerability of breast MRI in the prone position among pregnant patients, although most cases involved pregnant women in the first and second trimesters. In order to decrease any gravitational pressure from the belly, extra pillows were placed underneath the women to assist with pelvic lifting. In terms of diagnostic performance, diffusivity maps were useful in detecting nine out of 11 lesions and excluded malignancy in 14 high-risk patients; however, the maps were unable to detect two 7 mm lesions, as anticipated under the technical limitations of this modality [93]. Representative cases of unenhanced MRI in pregnant breast cancer patients are shown in Fig. 11 [94], highlighting the potential diagnostic advantages of this approach.

**Fig. 12:**
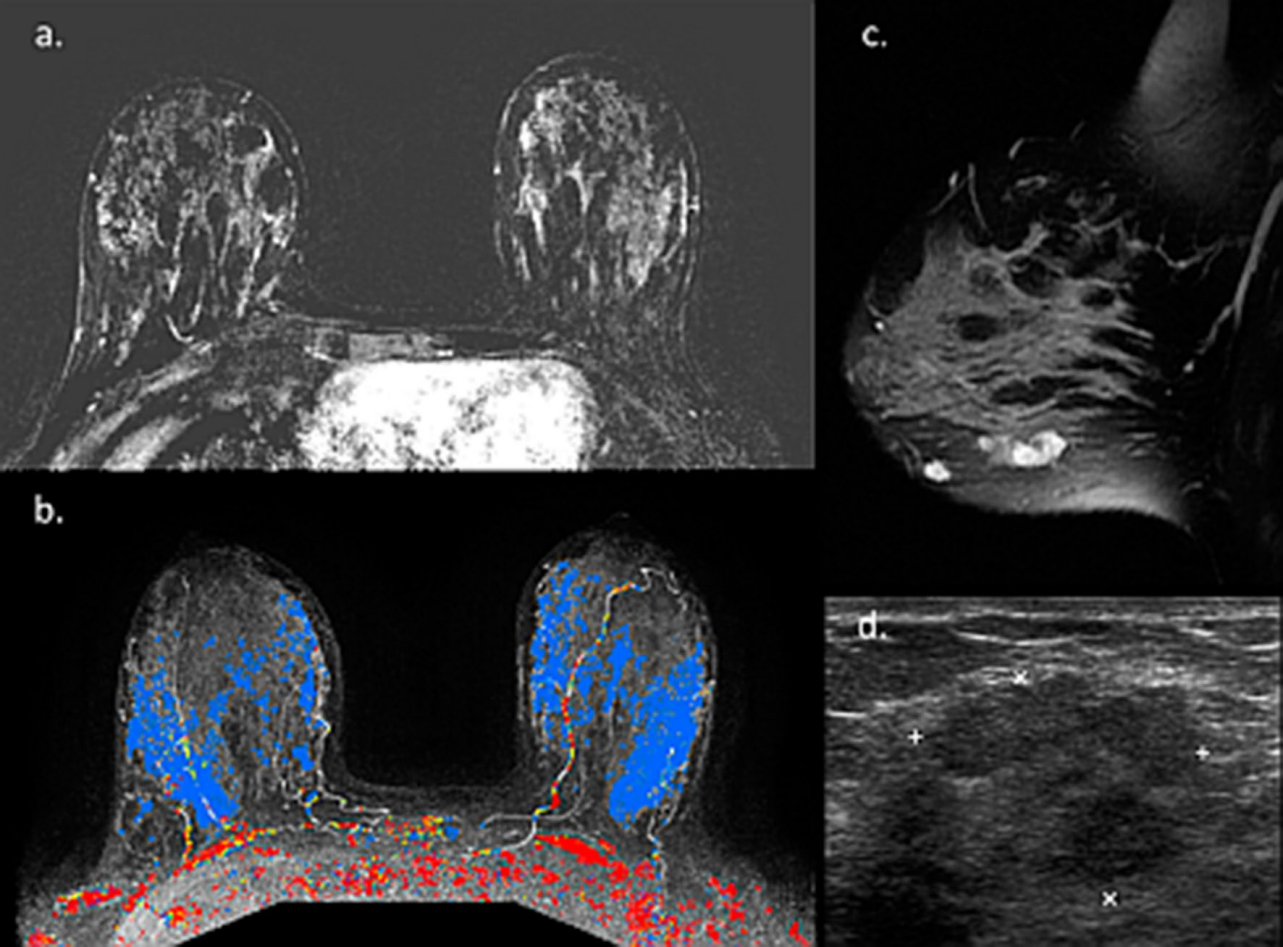
MRI of mucinous carcinoma during pregnancy. MR images of patient with left sided mucinous carcinoma, presented as a palpable mass during pregnancy are presented. The patient chose to undergo elected abortion. Subtraction DCE image reveals marked bilateral BPE (**a**) and CAD reveal bilateral diffuse benign-like coloring with progressive enhancement in time intensity curves (**b**) without evidence of the known underlying lesions in the left breast. However, the lesions, diagnosed on US (**d**), were clearly visible on sagittal T2-weighted fat-suppressed image (**c**)

An interesting and unusual case we encountered was of a pregnant patient with newly diagnosed mucinous breast carcinoma who underwent MRI prior to elected abortion. Pure mucinous carcinoma typically appears on MRI as a circumscribed mass with extremely high signal intensity on fat-saturated T2-weighted imaging and a benign-appearing persistent enhancement curve [94, 95]. As demonstrated in Fig. 12, the palpable lesion on the left breast was not detected on DCE and CAD images because of concurrence of its benign-like kinetic features and the marked surrounding BPE. Yet, the lesions were clearly visible on fat-suppressed T2-weighted images, therefore stressing the importance of acquiring broad protocol in diagnostic breast MRI.

Unenhanced diffusion MRI is also gaining recognition in the diagnostic workup of PABC for the systemic staging of pregnant patients, when the use of PET/CT is discouraged [95]. For this purpose, a whole-body MRI relying on DWI with background suppression (DWIBS) sequence has been applied [96]. This emerging MRI technique can provide non-invasive information regarding the extent of disease and distant metastasis and often provides diagnostic value that changes the patient management [97, 98].

### Lactation

Breast MRI is much more common during lactation, due to the fact that injection of gadolinium-based contrast agent is considered safe for administration [20]. Past studies evaluating the gadolinium excretion into breast milk revealed that less than 0.04% of the administered dose reaches the milk [99], and, of that amount, only 0.8% is actually absorbed by the infant [100]. Accordingly, some authors openly assert that lactating patients should not be advised to suspend breastfeeding at all, given that the risks associated with interrupting breastfeeding outweigh the negligible amount of contrast media [101]. More conservative approaches suggest the option of abstaining from breastfeeding for a period of 12–24 h if this is the preference of an informed mother [102]. Since the excretion of gadolinium to breast milk has been shown to reach its peak after approximately 4 h [103], if lactating patients have concerns about breastfeeding, the authors advise to pump and dump the milk with continuation of nursing after 6 h.

The main concern regarding the use of breast MRI during lactation does not stem from safety worries, but rather reservations regarding its uncertain diagnostic performance. In light of the increased BPE, there are concerns that it may potentially obscure the presence of the underlying tumor [27]. Several publications reported that despite increased surrounding BPE, high sensitivity was observed in known PABC cases that underwent DCE-MRI [40, 49–51].

**Fig. 13:**
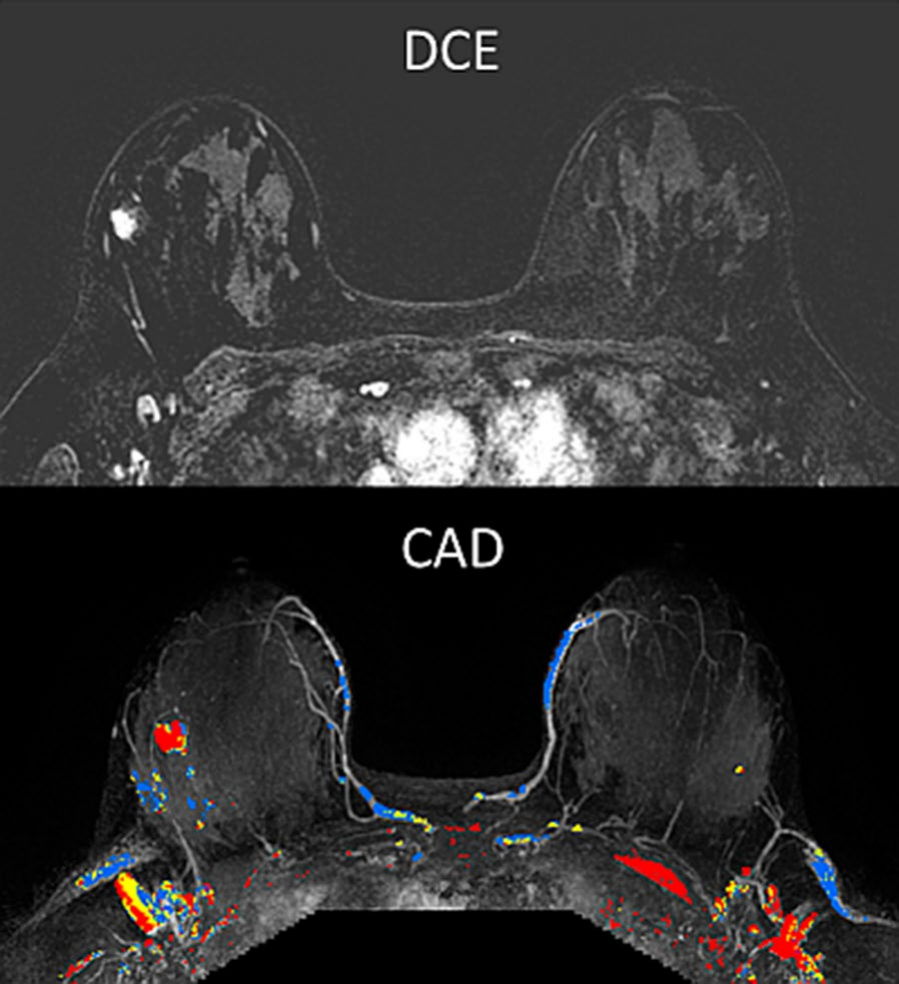
Preoperative MRI during lactation. Axial subtracted DCE-MRI and CAD images of a 37-year-old PABC patient, lactating for 3 months are shown. *Note*: DCE reveals the presence of a single small lesion in the right upper outer quadrant (12 mm IDC) with excellent conspicuity, comparing with the surrounding moderate BPE. The tumor exhibited the suspicious wash-out pattern (red) on CAD MIP image, while slight BPE exhibited persistence benign wash-in pattern (blue)

**Fig. 14:**
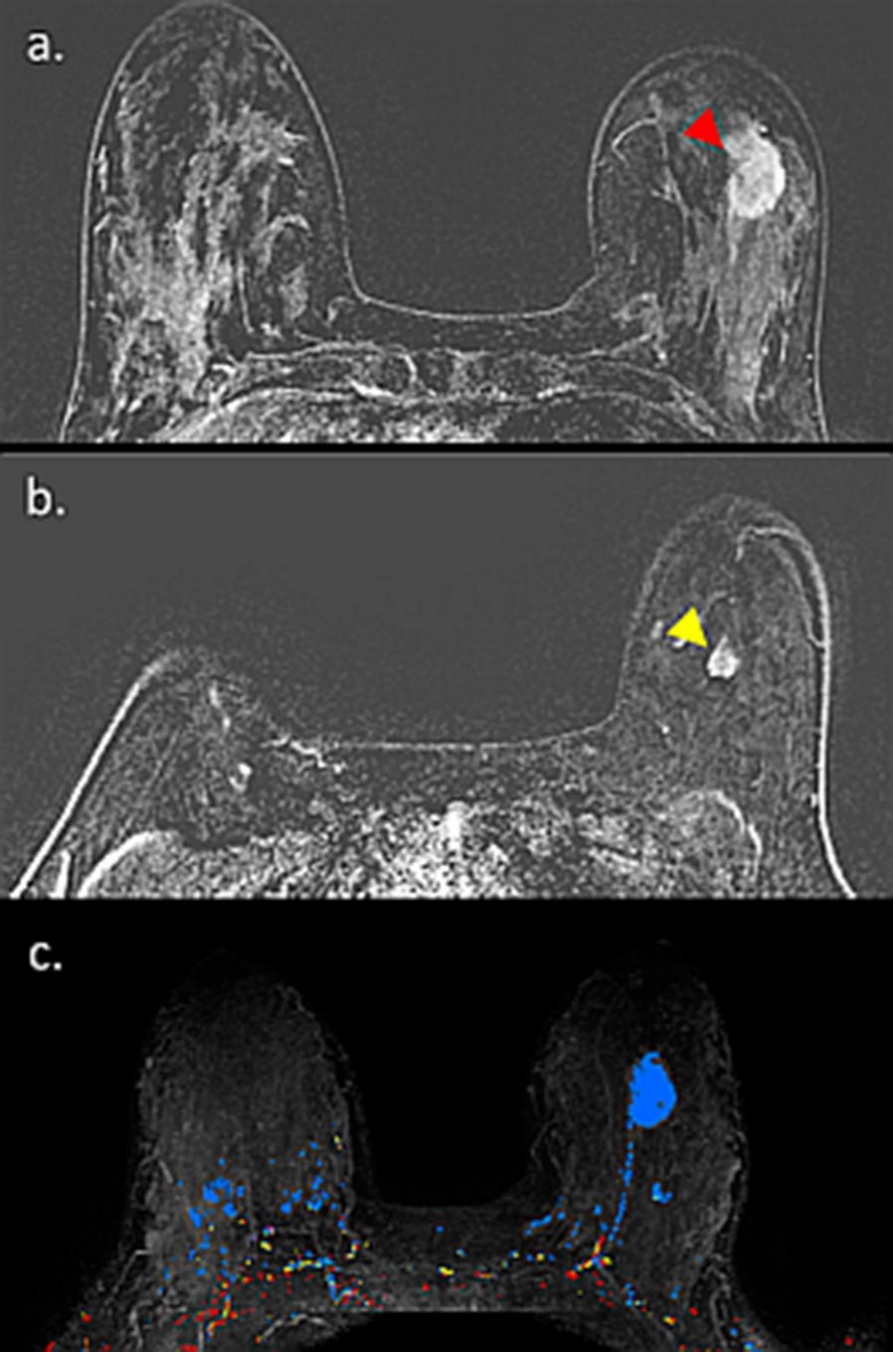
MRI of a complicated case of IDC with lactation changes. Axial subtracted DCE-MRI and CAD of lactating patient with newly diagnosed PABC, undergoing preoperative evaluation are shown. The tumor, 2.8 cm IDC in the left breast, is well visible on top of the surrounding BPE (**a**). Yet, additional ipsi-lateral enhancing 0.9 cm lesion is apparent (**b**). Upon focused US and US-guided biopsy, the second lesion came up to be adenosis and lactating changes on pathology. Interestingly, CAD depicted both lesions, as well as slight BPE in blue color, which corresponds to persistence wash-in pattern which usually represents a benign pattern (**c**)

Herein, we present an assembly of representative cases, illustrating the spectrum of appearance and persevered diagnostic capabilities of breast MRI, even in lactating patients. The first case is a pre-operative MRI of a PABC patient who presented with a palpable mass after 3 months of lactation. IDC was diagnosed using US-guided biopsy, and MRI reassured the existence of a solitary lesion on top of the surrounding BPE (Fig. 13). Occasionally, the diagnostic workup of known, newly diagnosed PABC can get complicated by simultaneous benign lactation-related findings, as shown by Fig. 14. In this patient, a preoperative MRI performed in a lactating patient revealed the known 2.8 cm IDC, as well as another enhancing 0.9 cm lesion which warranted focused US and biopsy to reveal adenosis and lactating changes on pathology. This case demonstrated that an argument regarding reduced specificity of breast MRI during lactation could be claimed.

**Fig. 15:**
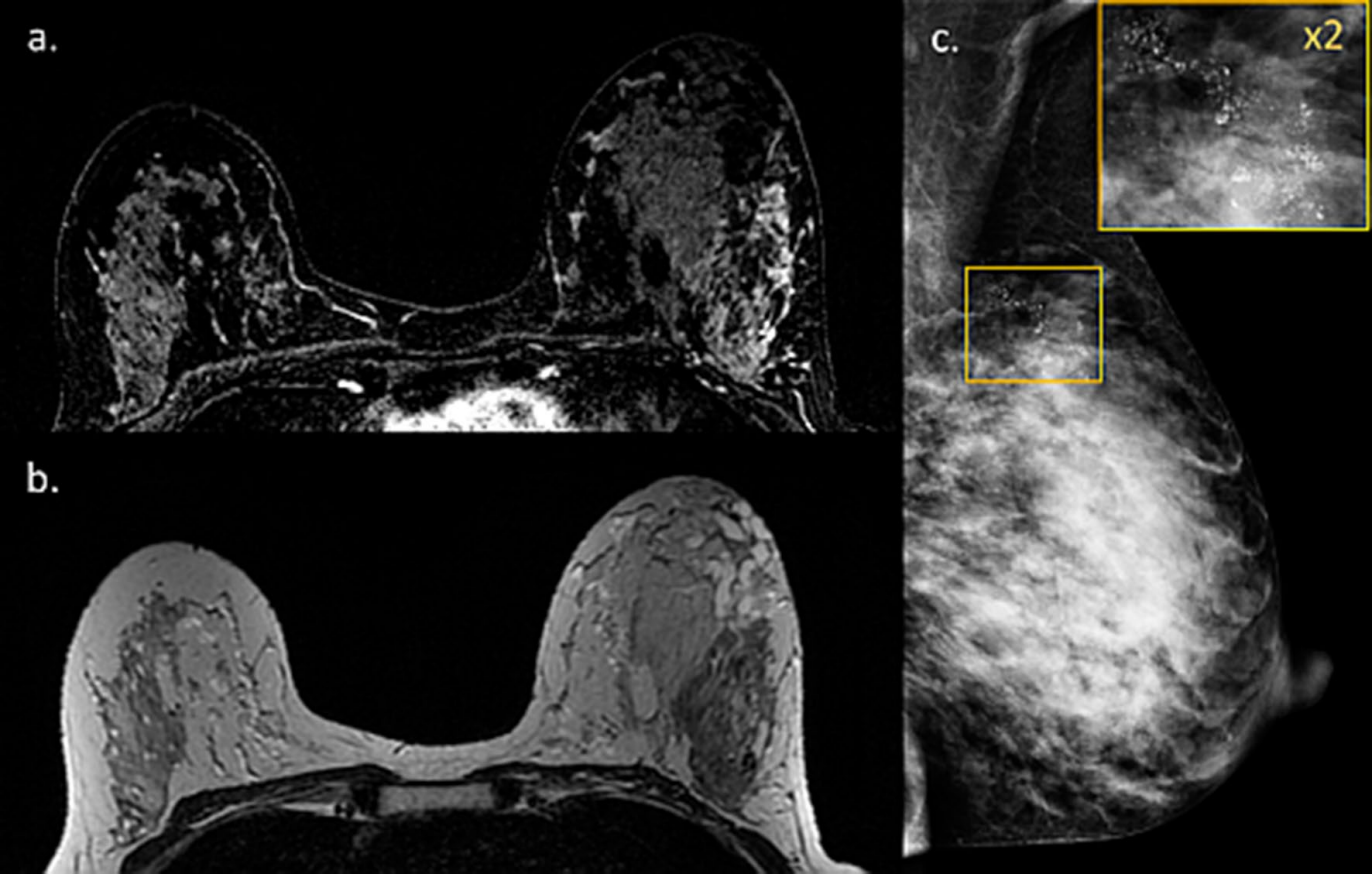
MRI of DCIS during lactation. Images of a 40-year-old PABC patient (lactating for 4 months) with DCIS confirmed on operation are presented. The patient underwent screening mammography with adjuvant breast US (not presented) due to the extremely dense breasts on mammography (BI-RADS D) (**c**). Mammography revealed suspicious pleomorphic micro-calcifications in segmental distribution along 6 cm in the left upper outer quadrant (**c**). Pre-operative MRI showed NME on top of the surrounding prominent lactation-induced BPE, typical for DCIS, in agreement with the calcifications location (**a**). Furthermore, an excellent tumor delineation was afforded by non-fat surpassed T2-weighted image, showing hypo-intense region in the tumor area (**b**)

**Fig. 16:**
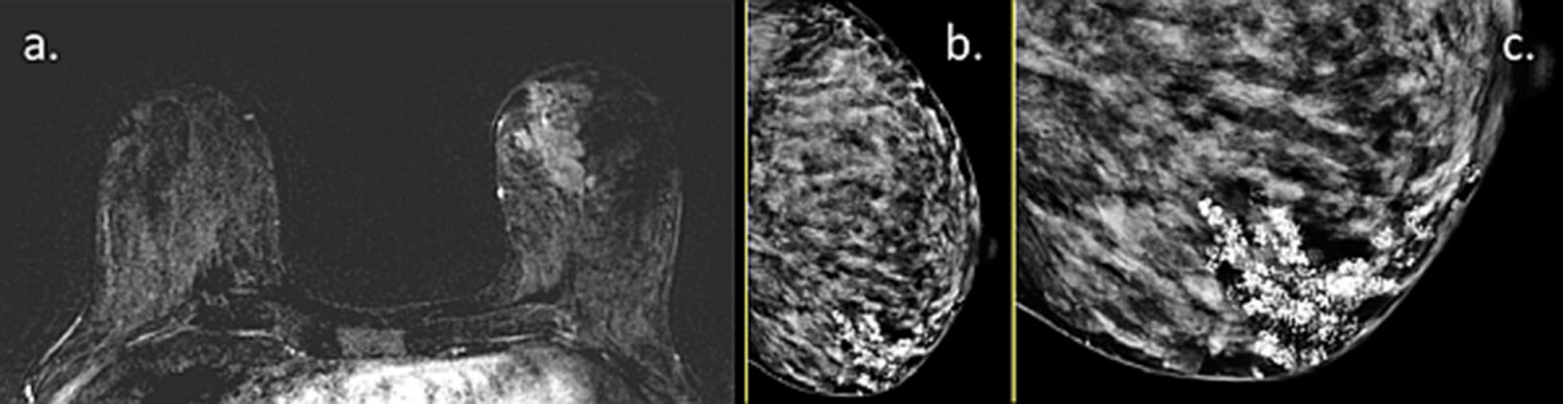
MRI of post-partum DCIS. Images of a 33-year-old PABC patient (lactating cessation a week prior to the MRI) diagnosed with left breast DCIS are presented. The patient palpate a lump in the left breast during the third trimester of pregnancy and underwent breast US (not presented) which depicted a benign appearing 9 mm oval mass. Upon follow-up 3 months later, post-partum, focal US depicted irregular mass with calcifications. Further diagnostic workup included US-guided biopsy, mammography which revealed extremely dense breast with segmental distribution of suspicious micro-calcifications in the inner-lower quadrant (**b** and zoomed image **c**), as well as breast MRI (**a**) which showed the characteristic NME of DCIS along 42 mm, in agreement with the mammographic findings, on top of the moderate background enhancement (BPE 2)

One noteworthy type of cancer that deserves a specific mention is ductal carcinoma in situ (DCIS), which often displays overlapping radiological and pathological features with lesions with uncertain malignant behavior [104]. Unlike invasive carcinomas that tend to present as a mass, DCE-MRI usually depicts DCIS as non-mass enhancement (NME) with a larger median span than mammography [105]. Taking into consideration the difficulty in unravelling BPE from NME [106], this casts doubt regarding the utility of breast MRI to detect DCIS during lactation could arise. Two representative newly diagnosed DCIS cases undergoing preoperative breast MRI during lactation are presented in Figs. 15 and 16. Both patients presented with palpable mass and mammography detected suspicious linear micro-calcifications in typical segmental distribution. DCE-MRI displayed NME in the tumor region, enhancing more vividly than the surrounding lactation-induced BPE. Interestingly, additional diagnostic value was provided by non-fat suppressed T2-weighted images, allowing better depiction of lesion morphology and margins (Fig. 15).

**Fig. 17:**
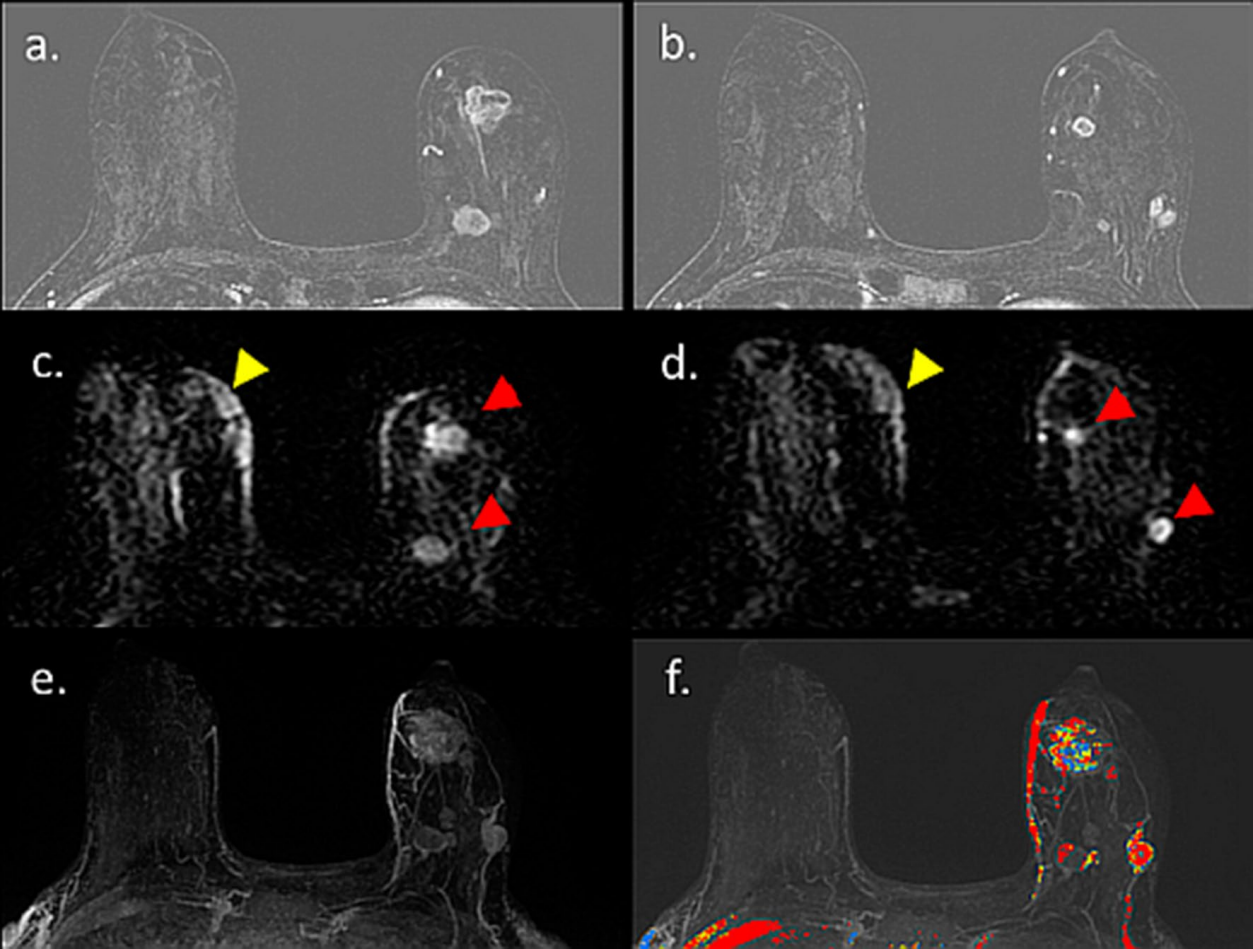
MRI of multi-centric IDC during lactation. MRI of a 40-year-old PABC patient (lactating for 18 months) is presented. The woman presented with a palpable left breast mass and was referred to pre-operative MRI upon IDC diagnosis. *Note*: Several multifocal tumor foci are shown on the subtracted DCE MRI of the left breast (red arrow heads), surrounded by a moderate BPE (grade 2) (**a**, **b**). In agreement, diffusion weighted images (b = 700) revealed restricted tumor regions (**c**, **d**), though incomplete fat saturation artifacts are also presented on the right breast (yellow arrow heads). The entire multifocal tumor distribution could be further appreciated on MIP images of DCE and CAD (**e** and **f**, respectively)

Occasionally, PABC can manifest as multi-centric carcinoma, which is difficult to fully estimate its extent using conventional imaging. A representative case of a lactating patient with newly diagnosed IDC which turned to comprise no less than seven distinct malignant ipsi-lateral lesions is presented in Fig. 17, highlighting the ability of DCE-MRI, as well as unenhanced DWI to portray the entire extent of disease. With that regards, a recent comparative study investigated tumor conspicuity in DCE-MRI and unenhanced DTI protocol among lactating patients with PABC [51]. On DCE-MRI, because of the marked BPE, tumor conspicuity was reduced by 60% as compared to non-lactating controls. On the contrary, an additional 138% increase in tumor conspicuity on DTI compared with DCE was observed, underscoring a clear advantage for unenhanced MRI to operate in the setting of lactation-induced BPE.

**Fig. 18:**
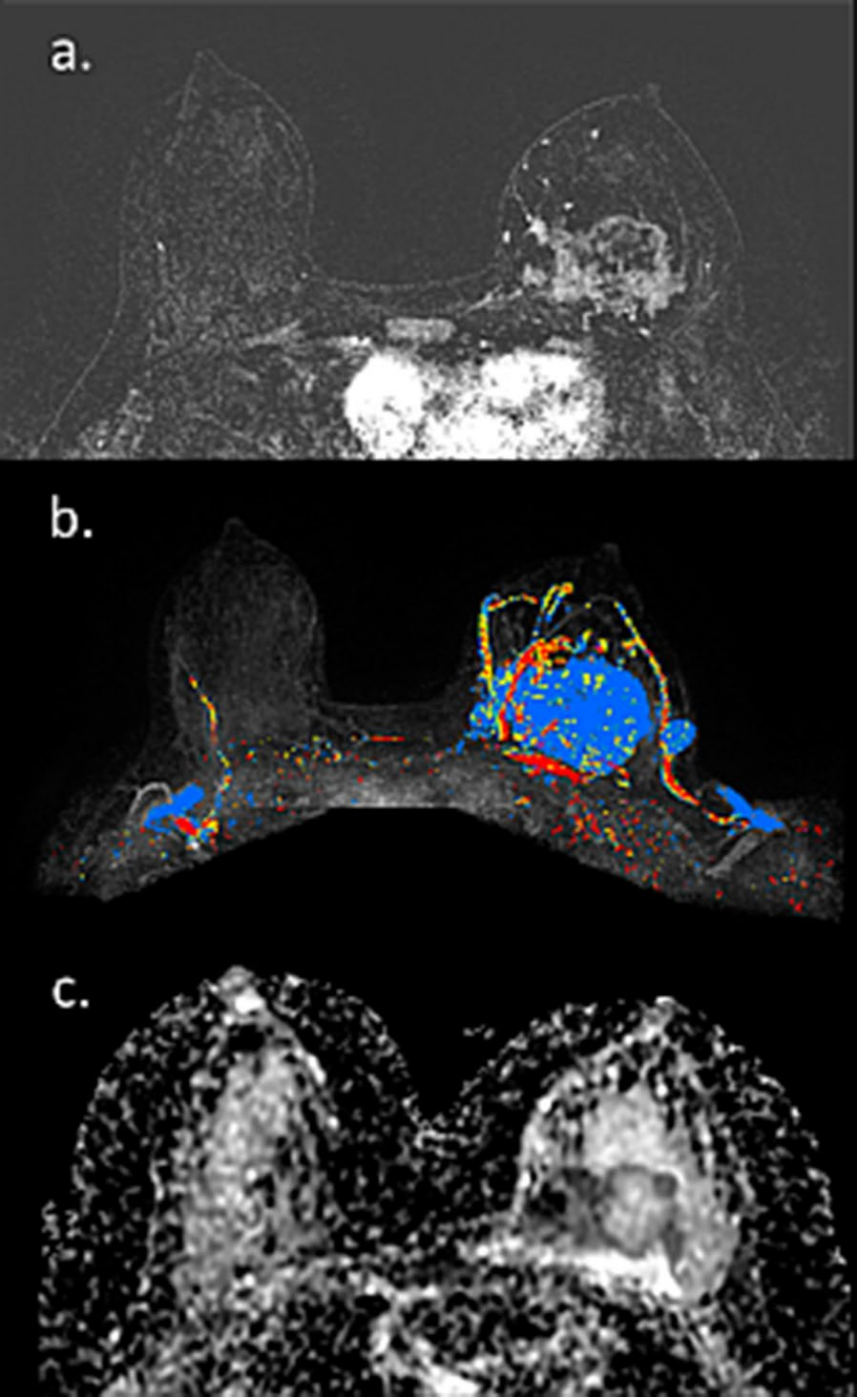
MRI of delayed diagnosed IDC during lactation. Axial subtraction DCE-MRI, CAD and ADC map of a 25-year-old PABC patient lactating for 8 months, with a 6.7 cm triple negative IDC on the right breast are presented. The massive lesion exhibited with an irregular rim enhancement concordant with triple negative IDC and is clearly visible on top of the surrounding mild BPE (**a**), with mostly persistent enhancement kinetic pattern (**b**) and decreased ADC values in margins of the lesions, with increased diffusivity in the central necrotic region (**c**)

**Fig. 19:**
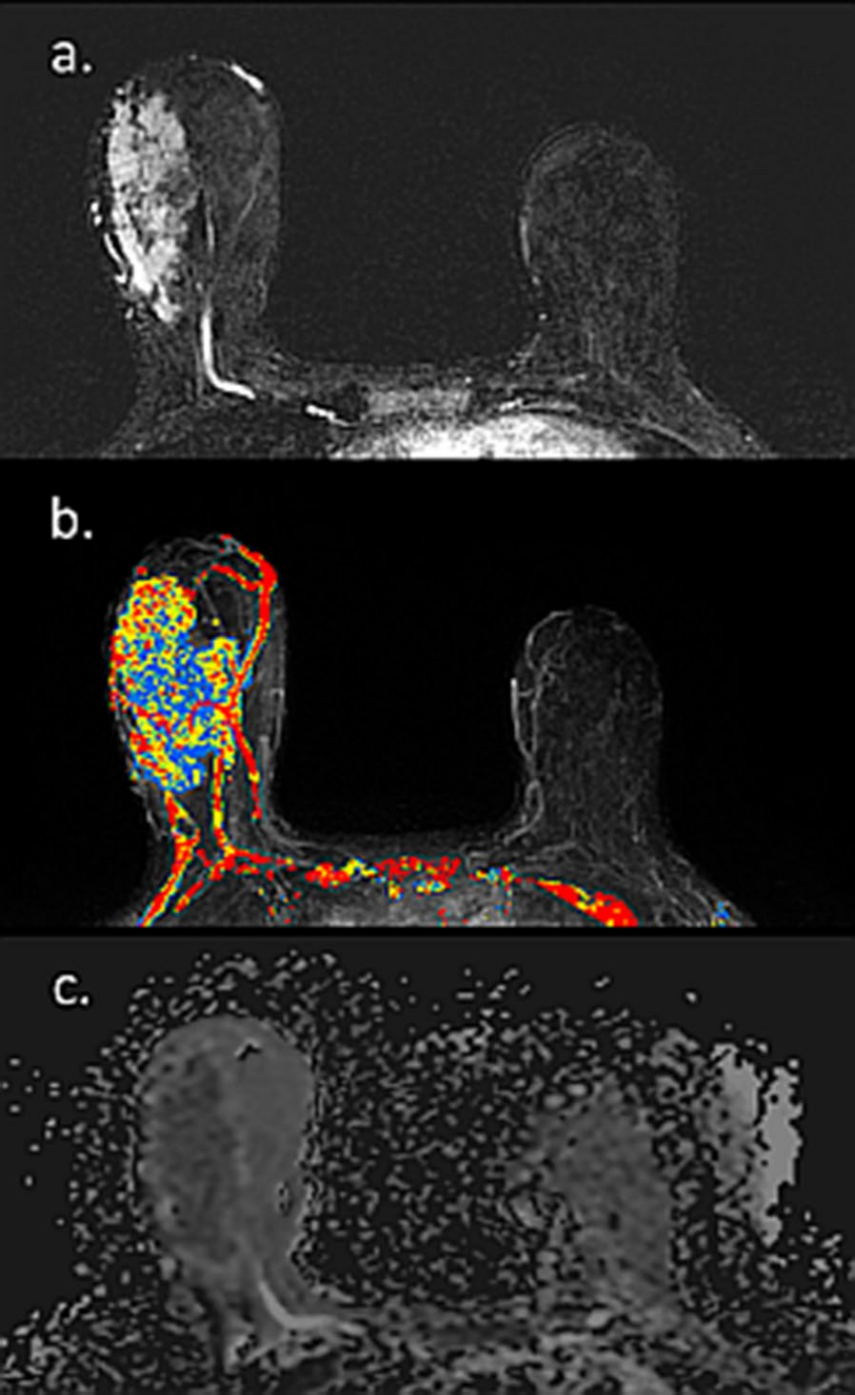
MRI of delayed diagnosed IDC during lactation. Axial subtraction DCE-MRI, CAD and ADC map of a lactating patient with a 7 cm IDC on the right breast are presented. The huge lesion exhibited vivid enhancement as compared to the mild BPE (**a**), with heterogeneous kinetic features (**b**) and decreased ADC values as compared with the surrounding parenchymal diffusivity (**c**)

Since PABC is often a delayed diagnosis, it is associated with more advanced tumor size at the time of diagnosis compared to non-PABC [107] and eventually may lead to increased rates of mastectomy as the treatment of choice [108]. Therefore, it is not uncommon to encounter a large PABC lesion occupying a high portion of the breast size, as demonstrated in Figs. 18 and 19. These images show the complete extent of the enormous tumors which are clearly depicted on both DCE as well as on unenhanced DWI.

### Post-weaning

**Fig. 20:**
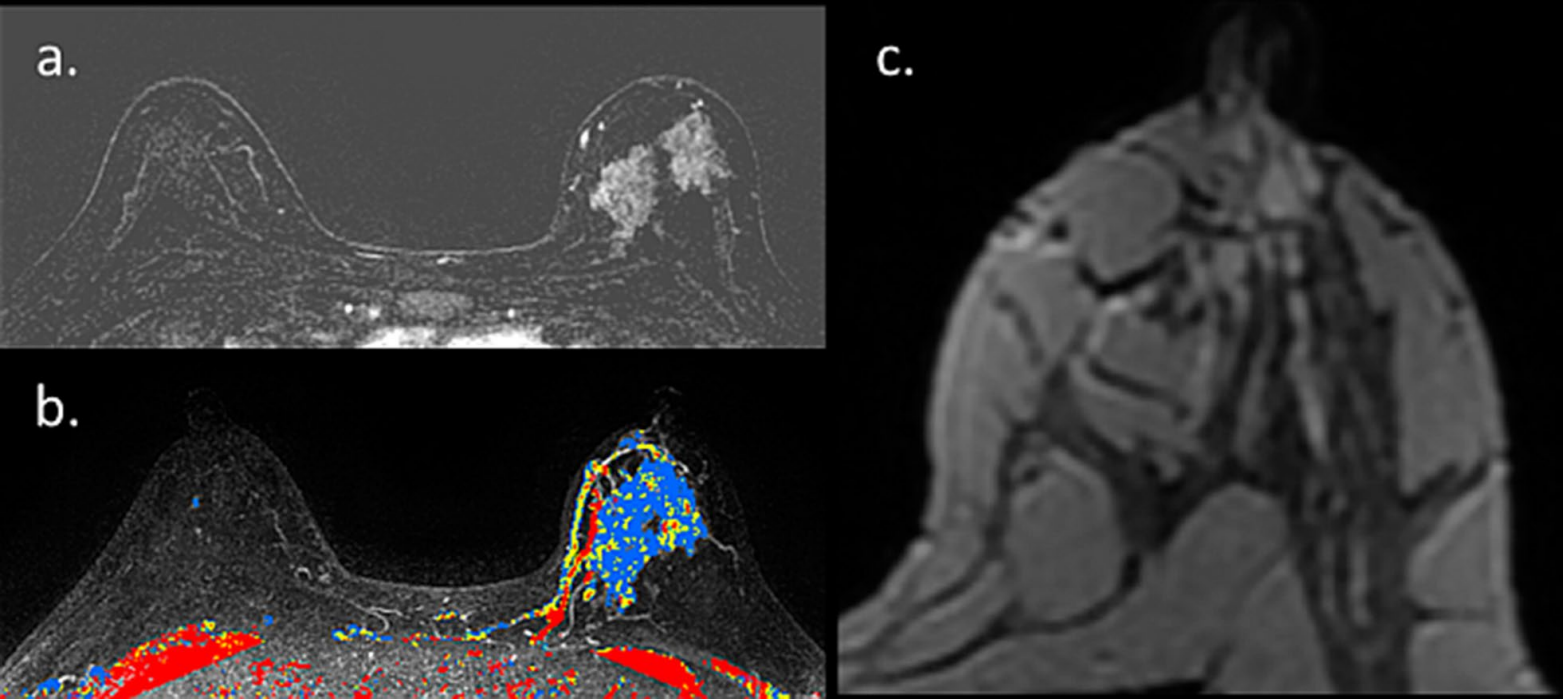
MRI of breast cancer diagnosed post-weaning. Pre-operative MR images of 42-year-old patient with DCIS, newly diagnosed to months after cessation of breastfeeding are presented. Subtraction DCE (**a**) and CAD (**b**) reveal large nonmass enhancing lesion in the left breast, on top of minimal BPE. Non-fat surpassed T2-weighted image at the height of the nipple reveal high signal ducts in the sub-areolar region (**c**), representing the transformation of the breast from lactation to involution

Considering the difficulty of interpretation of DCE-MRI with marked BPE and the high likelihood of lactation-related benign entities, some authors suggest that it may be reasonable to delay the examination until several months after weaning to minimize false-positive results that may lead to unnecessary biopsies [26]. Screening MRI was once recommended in the breastfeeding period for “women who are at very high-risk for breast cancer” [7], or within the first 6 months postpartum [109]. Others suggested waiting until 3 months after cessation of breastfeeding since the imaging changes should resolve by this time-span following lactation cessation [6]. Recently, the ACR guidelines recommended resuming MRI screening for patients over 30 years old if breastfeeding is continued for more than 6 months. Otherwise, the ACR recommends resuming annual high-risk screening MRI 6–8 weeks following cessation of breastfeeding [20]. All in all, despite the variance in the literature, based on our institutional experience, the authors advocate not to postpone pre-operative MRI of newly diagnosed lactating PABC patient. Usually, from the beginning of the diagnostic work-up and until pathological confirmation of the cancer, the patients often discontinue nursing, and even this interlude period of 1–2 weeks may be sufficient to decrease the level of BPE. Among patients who are diagnosed with breast cancer post-weaning, lactation-related BPE is no longer expected and the tumor can be clearly viewed by DCE (Fig. 20).

## Summary and outlook

In light of the marked physiological changes that the breast undergoes during pregnancy and lactation, clinical and radiological evaluation of the breast becomes extremely challenging. Considering the high incidence of gestational-associated benign breast entities, it is no surprise that PABC is often a delayed diagnosis [110]. The delay could be attributed to either the patient, if they postpone seeking medical evaluation, the physician, if they provide a false-negative clinical assessment of the symptomatic breast, or an imaging-related delay, via a false-negative radiological evaluation [111]. Ultimately, PABC is typically diagnosed only after clinical symptoms arise, most commonly as a large palpable mass [112]. Considering that PABC’s prognosis is not inferior from that of non-PABC when adjusted for stage and age [113], it appears that the delay in diagnosis, rather than the gestational state and associated overexpressed vascular, hormonal and growth-factor mediators [114], is responsible for its poor prognosis. This demonstrates the unmet need to adapt new screening strategies for high-risk populations during this period [20, 109], as well as to develop and utilize advanced imaging tools for achieving early diagnosis.

While there is wide agreement that US should be the first-line modality for breast imaging during pregnancy and lactation, and that mammography may have a supplementary additive role, the role of MRI remains controversial in the diagnostic work-up of PABC. In this pictorial review, we have illustrated how gestational-related physiological and benign processes are translated to MRI. Moreover, we have demonstrated the promising utility of unenhanced MRI to serve as a standalone breast imaging modality during pregnancy, and the more established utility of both contrast enhanced and unenhanced breast MRI during lactation. Specifically, it appears that since most cases of PABC reach the radiological work-up with a large palpable mass, the opportunity to facilitate an earlier diagnosis of PABC could be found among high-risk patients and BRCA mutation carriers, which account for up to 35% of PABC cases [115]. In this population, action should be taken to investigate whether screening MRI can detect PABC with asymptomatic disease.

Unenhanced breast MRI using DWI variants has shown great strides to serve as a possible cost-effective, fast, and clinically effective alternative to DCE [116]. Nevertheless, several factors are still holding it from being fully integrated into daily practice [117]. Technically, breast DWI is prone to eddy currents, geometrical and intensity distortions, and echo planar imaging ghosts artifacts [93]. Clinically, lower sensitivity of breast DWI was noted in cases of sub-centimeter lesions [118, 119], as well as in NME lesions [120]. To overcome these drawbacks, several strategies were recently attempted in order to provide robustness to artifacts and improve image quality [121–125]. Spatial resolution was also improved by reaching up to sub-millimeter pixel resolution [126–128], eventually allowing for visibility of higher lesions [129] and greater morphological concordance between DWI and DCE [130]. Thus, the authors foresee an encouraging future for breast DWI in general, and in particular with PABC.

**Fig. 21:**
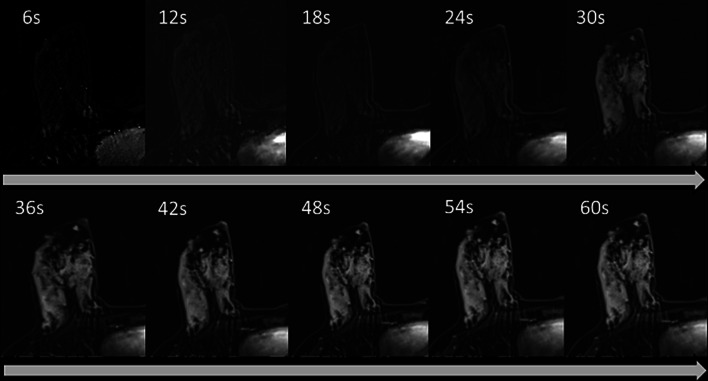
Ultrafast DCE-MRI of the lactating breast. Subtracted ultrafast DCE-MRI of the right breast of a healthy lactating patient (34 years old, lactation duration 18 months) are presented. Using grasp-vibe, a compressed-sensing technique, acquisition of ten consecutive T1-weighted images in the first minute post injection was enabled. *Note*: the prominent lactation-induced BPE (grade 3) appears only in the fifth acquired T1 image, approximately after 30 s, and further enhances in the following phases

For DCE, it is safe to assume that during pregnancy it would remain unutilized. During lactation, however, the role of DCE may expand, possibly due to the implementation of novel acquisition schemes that may allow better separation between enhancing lesions and BPE. In recent years, developments in accelerated MRI using the application of compressed sensing [131] have allowed the faster acquisition of MRI data. This relies on exploiting sparsely under-sampled k-space in peripheral regions while continuously sampling the k-space center to enable high temporal resolution with preserved spatial resolution. Several sparse methods have been integrated to MRI protocols, including time-resolved angiography with stochastic trajectory (TWIST) [132] and golden-angle radial sparse parallel (GRASP) [133]. Optimization of sparse techniques to breast MRI has promoted the novel approach of ultrafast DCE with temporal resolution of less than 10 s during the initial wash-in phase, compared with a standard temporal resolution of up to 2 min in conventional MRI [134]. Analysis of the wash-in kinetics has been found to provide valuable information for lesion characterization [134–139] and since BPE usually exhibits slow early enhancement slope and persistent delayed enhancement [140], ultrafast sequence might therefore be suitable for early visualization of malignant lesions with minimization of lactation-induced BPE [141]. The accumulation of BPE along the early phases of wash-in during ultrafast breast DCE of healthy lactating patients is demonstrated in Fig. 21. Altogether, there is a clinical necessity of further studies on larger cohort of patients to evaluate the role of breast MRI during pregnancy and lactation, and in particular as a screening tool among high-risk populations during this period.

## Conclusions

During pregnancy and lactation, the breast experiences substantial changes in morphology and function that affect its imaging properties and may reduce the visibility of concurrent pathological processes. Moreover, the high incidence of benign, gestational-related entities may further add complexity to the clinical and radiological evaluation of the breast during this period. Consequently, PABC is often a delayed diagnosis that carries a poor prognosis. Despite currently being underutilized, this state-of-the-art pictorial review illustrates how technical advances and new clinical evidence support the use of unenhanced breast MRI during pregnancy and both unenhanced and DCE during lactation. These modalities serve as effective supplementary options in the diagnostic work-up of PABC, especially among high risk populations, with the aim to facilitate an earlier diagnosis.

## Data Availability

Not applicable.

## Abbreviations

ACR: American college of radiology
ADC: Apparent diffusion coefficient
BPE: Background parenchymal enhancement
CAD: Computer aided diagnosis
DTI: Diffusion tensor imaging
DWI: Diffusion-weighted imaging
DCIS: Ductal carcinoma in situ
DWIBS: DWI with background suppression
DCE: Dynamic-contrast enhanced
GRASP: Golden-angle radial sparse parallel
GM: Granulomatous mastitis
IVIM: Intra-voxel incoherent motion
IDC: Invasive ductal carcinoma
MRI: Magnetic resonance imaging
NME: Non-mass enhancement
PABC: Pregnancy associated breast cancer
TWIST: Time-resolved angiography with stochastic trajectory
